# The Hypoxia–Long Noncoding RNA Interaction in Solid Cancers

**DOI:** 10.3390/ijms22147261

**Published:** 2021-07-06

**Authors:** Seung Wan Son, Ba Da Yun, Mun Gyu Song, Jin Kyeong Lee, Soo Young Choi, Hyo Jeong Kuh, Jong Kook Park

**Affiliations:** 1 Department of Biomedical Science, Research Institute for Bioscience & Biotechnology, Hallym University, Chunchon 24252, Korea; miyanae@naver.com (S.W.S.); dbsbada@naver.com (B.D.Y.); smgdd@naver.com (M.G.S.); mous96@naver.com (J.K.L.); sychoi@hallym.ac.kr (S.Y.C.); 2 Department of Medical Life Sciences, College of Medicine, The Catholic University of Korea, Seoul 06591, Korea; hkuh@catholic.ac.kr

**Keywords:** hypoxia, hypoxia-inducible factors, HIF, long noncoding RNA, cancer

## Abstract

Hypoxia is one of the representative microenvironment features in cancer and is considered to be associated with the dismal prognosis of patients. Hypoxia-driven cellular pathways are largely regulated by hypoxia-inducible factors (HIFs) and notably exert influence on the hallmarks of cancer, such as stemness, angiogenesis, invasion, metastasis, and the resistance towards apoptotic cell death and therapeutic resistance; therefore, hypoxia has been considered as a potential hurdle for cancer therapy. Growing evidence has demonstrated that long noncoding RNAs (lncRNAs) are dysregulated in cancer and take part in gene regulatory networks owing to their various modes of action through interacting with proteins and microRNAs. In this review, we focus attention on the relationship between hypoxia/HIFs and lncRNAs, in company with the possibility of lncRNAs as candidate molecules for controlling cancer.

## 1. Introduction

Hypoxia is an intrinsic characteristic of solid cancers and is perceived as an impediment towards efficient cancer treatments. In-depth knowledge of the hypoxia-mediated signaling pathway is vital for the establishment of novel treatment strategies against cancer. Long noncoding RNAs (lncRNAs) have been recognized as essential regulators of cellular signaling pathways and as therapeutic targets in cancer. This review highlights the inter-linkage between hypoxia and lncRNAs, together with the feasibility of exploiting lncRNAs for the treatment of cancer.

### 1.1. Hypoxia and Hypoxia-Inducible Factors

The intracellular signaling pathways that respond to hypoxia are mainly regulated by hypoxia-inducible factors (HIFs) [1]. Oxygen-sensitive HIF-1α and HIF-2α subunits heterodimerize with HIF-1β, a constitutively expressed subunit, to form HIF-1 and HIF-2 transcription factors, respectively. The ubiquitination and proteasomal degradation of HIF-1α and HIF-2α are decreased under hypoxia [1]. HIF-3α is an additional alpha subunit and is generally known to suppress HIF-dependent regulation of target genes via competition with HIF-1α and HIF-2α [2]. However, depending on the type of transcription isoform, HIF-3α can serve as an oncogenic factor by promoting cell proliferation, invasion, and metastasis [3]. Additionally, it has been noted that hypoxia-mediated signaling is regulated in a HIF-independent manner [4,5]. Additionally, the expression and activity of HIF-1α and HIF-2α can be controlled independently of hypoxic conditions [6,7].

### 1.2. Hypoxia and Cancer

A broad spectrum of cellular signaling events are influenced by hypoxia, leading to the malignant progression of cancer. HIF-1 can upregulate and downregulate the level of myeloid cell leukemia 1 (*MCL1*) and BH3-interacting domain death agonist (*BID*), respectively, leading to the protection of cells from apoptotic cell death [8,9]. In addition, activation of the p53 pathway is antagonized by HIF-1 and HIF-2 [10,11]. Hypoxia also activates the epithelial-to-mesenchymal transition (EMT) process and cancer stemness, eventually promoting cancer aggressiveness and metastasis [12,13,14,15,16]. In terms of therapeutic resistance, several cellular factors and events including anti-apoptotic/survival factors, EMT, and stemness are associated with a reduction in the sensitivity of cells to cancer treatments [17,18]. Therefore, hypoxia is considered as one of the causes of drug resistance in cancer. Another well-known effect of hypoxia includes the augmentation of angiogenesis. The expression of angiogenesis factors, such as vascular endothelial growth factor (VEGF), is increased by hypoxia in cancer cells and other cellular components in the microenvironment, such as endothelial cells and cancer-associated fibroblasts (CAFs), thereby increasing the metastatic potential of cancer [19,20,21].

Moreover, hypoxia induces several enzymes, such as glucose transporters and pyruvate dehydrogenase kinases, that reprogram cancer cell metabolism from oxidative phosphorylation to glycolysis. The production of lactic acids through hypoxia-mediated glycolysis creates an acidic microenvironment in cancers. This metabolic reprogramming consequently supports multiple cellular processes, such as cell survival, stemness, angiogenesis, and metastasis, and causes drug resistance [22,23,24,25,26,27]. Hypoxia diminishes anticancer immunity as well. For example, the uptake of antigens by dendritic cells is inhibited by hypoxic conditions [28,29]. Immune response can be subdued by regulatory T cells (Tregs), which are capable of producing immune-suppressive cytokines and inhibiting the activity of effector cells, such as T cells and natural killer cells [30,31,32]. Hypoxic cancer cells can upregulate C-C motif chemokine ligand 28 (*CCL28*) levels via HIF-1α, stimulating the recruitment of Tregs into the tumor microenvironment and allowing cancer cells to avoid immune surveillance [33]. Hypoxia also contributes to immune tolerance via transforming growth factor β (*TGF-β*)-mediated enrichment of Tregs in cancer [34].

### 1.3. LncRNAs

LncRNAs have been shown to regulate gene expression at multiple levels. As an illustration, lncRNA HOTAIR can mediate histone modifications in target genes by recruiting chromatin-modifying enzymes, thus being able to promote malignant properties such as EMT [35]. LncRNA PANDA directly binds to nuclear transcription factor Y subunit alpha (*NFYA*), restricts the expression of pro-apoptotic genes, and desensitizes cells to doxorubicin-induced apoptotic cell death [36], indicating that the interaction of lncRNAs with transcription factors regulates gene transcription as well. It has been also demonstrated that lncRNAs modulate the stability and activity of proteins, thereby affecting the progression of cancers [37,38]. Further, one of the documented activities of lncRNAs is to serve as competitive endogenous RNAs (ceRNAs) by sequestrating microRNAs (miRNAs). By molecularly sponging miRNAs, lncRNAs can limit and increase the expression of miRNAs and target messenger RNAs (mRNAs) of miRNAs, respectively [39]. However, it is noteworthy that the function of ceRNAs remains a controversial issue. For example, it was reported that the alteration of lncRNA expression within a physiological range is insufficient to change miRNA activities [40,41], suggesting the requirement of an improved understanding of ceRNA mechanisms.

## 2. LncRNAs Controlled by Hypoxia and HIFs

Although lncRNAs whose expression is affected by hypoxia/HIFs can exert diverse cellular effects, lncRNAs are subdivided into five groups in an attempt to present the crucial function of each lncRNA.

### 2.1. LncRNAs Regulating Cell Survival and Apoptosis

#### 2.1.1. H19

**Figure 1:**
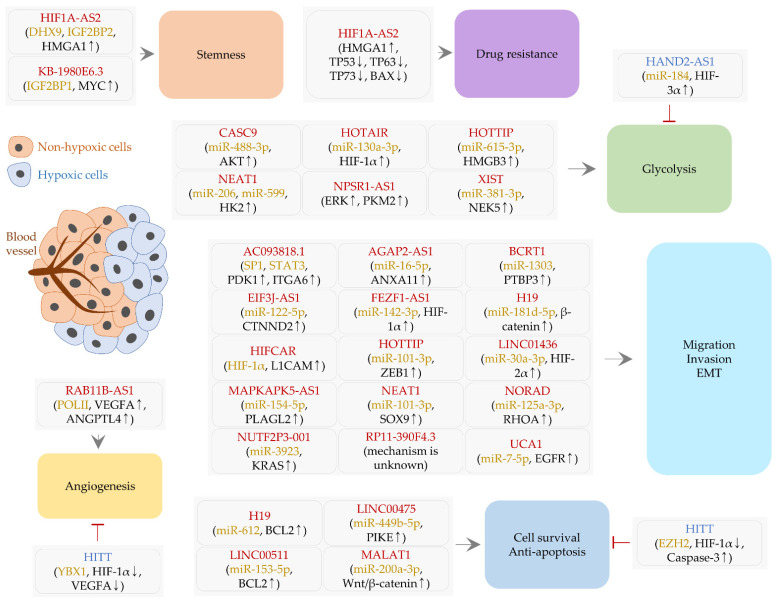
LncRNAs regulated by hypoxia and HIFs. Both oncogenic (red) and tumor-suppressive (blue) lncRNAs are presented in rounded rectangles. Round brackets denote proteins and miRNAs (orange) that directly interact with lncRNAs and then downstream cellular factors (black) consequently affected by lncRNA-protein/miRNA interactions. Positive regulation is shown by an arrow. An inhibitory effect is designated by a perpendicular line.

Several studies demonstrated that miRNA-612 (miR-612) exerts tumor-suppressive effects through targeting multiple anti-apoptotic genes, such as bromodomain-containing protein 4 (*BRD4*), AKT serine/threonine kinase 2 (*AKT2*), and NIN1/PSMD8 binding protein 1 homolog (*NOB1*) [42,43,44]. Moreover, miR-612 can negatively regulate the expression of B-cell CLL/lymphoma 2 (*BCL2*) via interacting with the 3′ untranslated region (3′ UTR) [45]. In this study, it was further shown that H19 is induced by HIF-1α and renders miR-612 inactive, resulting in the upregulation of BCL2. Moreover, the knockdown of HIF-1α significantly restrains the growth of cholangiocarcinoma in vivo, along with miR-612 upregulation and BCL2 downregulation [45] (Figure 1 and Table 1).

#### 2.1.2. HITT

Enhancer of zeste homolog 2 (*EZH2*), a histone methyltransferase, is one of the subunits of polycomb repressive complex 2 (*PRC2*) and transcriptionally perturbs the expression of target genes by catalyzing histone H3 methylation [77]. It was recently revealed that the level of HITT is downregulated by hypoxia. Moreover, HITT was found to interact with EZH2 proteins and guide them to the promoter of HIF-1α, exhibiting a deterrent effect on HIF-1α transcription. The overexpression of HITT inhibits HIF-1α levels and increases caspase-3 activation and apoptotic cell death under hypoxia [57] (Figure 1 and Table 1).

#### 2.1.3. LINC00475

In glioblastoma, it was identified that miR-449b-5p targets phosphatidylinositol 3-kinase enhancer (*PIKE*, also known as ArfGAP with GTPase domain, ankyrin repeat and PH domain 2 (*AGAP2*)) [63], which possesses an anti-apoptotic activity [78,79]. LINC00475 can upregulate PIKE by impeding miR-449b-5p activities. The knockdown of LINC00475 induces apoptotic cell death in vitro and restricts the growth of glioblastoma in vivo [63] (Figure 1 and Table 1). Accumulating evidence reveals that miR-449b-5p serves as a tumor suppressor by suppressing various cellular factors, such as yin and yang 1 (*YY1*), cell-cycle related and expression-elevated protein in tumor (*CREPT*), and Wnt family member 2B (*WNT2B*) [80,81,82]. Given that stemness can be facilitated by YY1, CREPT, and Wnt/β-catenin signaling [83,84,85], LINC00475 may also contribute to increasing the stemness property of cancer cells.

#### 2.1.4. LINC00511

It has been noticed that LINC00511 facilitates migration and invasion in several types of cancer, including breast, lung, and pancreatic cancer [86,87,88]. LINC00511 was also reported to play an oncogenic role via upregulating and downregulating nuclear factor I/A (*NFIA*) and interleukin 24 (*IL-24*), respectively, in colorectal cancer [89,90]. Moreover, it was discerned that LINC00511 is transcriptionally activated by HIF-1α, blocks the function of miR-153-5p, and supports cell survival in colorectal cancer [64]. Since miR-153-5p targets BCL2 [91], LINC00511 may exert an anti-apoptotic activity, at least partly through augmenting BCL2 levels (Figure 1 and Table 1).

#### 2.1.5. MALAT1

Depending on the cancer type, MALAT1 functions as an oncogenic or a tumor-suppressive factor. For example, MALAT1 prohibits the lung metastasis of breast cancer [92]. By contrast, MALAT1 accelerates cell proliferation and metastasis via stimulating autophagy in pancreatic cancer [93]. In hepatocellular carcinoma, MALAT1 can suppress the induction of apoptosis via triggering PI3K/AKT signaling [94]. Moreover, a recent study demonstrated that hypoxia stimulates MALAT1 expression and that the knockdown of this lncRNA increases miR-200a-3p levels and induces apoptosis in hepatocellular carcinoma cells under hypoxic conditions [66]. Given that miR-200a-3p acts as an apoptosis-promoting miRNA by inactivating Wnt/β-catenin signaling [95], MALAT1 may block apoptotic cell death through the miR-200a-3p/Wnt/β-catenin signaling axis (Figure 1 and Table 1).

### 2.2. LncRNAs Affecting Cell Migration, Invasion, and EMT

#### 2.2.1. AC093818.1

AC093818.1 (also referred to as IHAT) binds to Sp1 transcription Factor (*SP1*) and signal transducer and activator of transcription 3 (*STAT3*), thereby mediating transcriptional activation of pyruvate dehydrogenase kinase 1 (*PDK1*). Therefore, AC093818.1 can accelerate cell migration and invasion in vitro and metastasis of gastric cancer in vivo [96]. Recently, whole transcriptome sequencing revealed that AC093818.1 is one of the lncRNAs upregulated by hypoxia in triple-negative breast cancer [46]. It was consistently observed that AC093818.1 promoted the lung metastasis of breast cancer in vivo. Mechanistically, AC093818.1 was proven to positively regulate the expression of PDK1 and integrin subunit alpha 6 (*ITGA6*) [46]. Since SP1 can positively control the level of ITGA6 [97], AC093818.1 may regulate ITGA6 via SP1 (Figure 1 and Table 1).

#### 2.2.2. AGAP2-AS1 and EIF3J-AS1

It was confirmed that both AGAP2-AS1 and EIF3J-AS1 are induced by hypoxia in hepatocellular carcinoma [47,50]. AGAP2-AS1 promotes cell migration, invasion, and the EMT process by sequestering miR-16-5p that directly targets annexin A11 (*ANXA11*), which is able to activate AKT. Furthermore, it was noticed that the overexpression and downregulation of AGAP2-AS1 promoted and reduced lung metastasis of cancer cells in vivo [47]. In the case of EIF3J-AS1, this lncRNA inactivates miR-122-5p to augment the level of catenin delta 2 (*CTNND2*). Whereas EIF3J-AS1 reinforces cell migration and invasion, hypoxia-induced cell migration and invasion are weakened in EIF3J-AS1-depleted cells [50] (Figure 1 and Table 1). CTNND2 has been discerned to accelerate migration, invasion, and metastasis through triggering the Wnt/β-catenin and Rac family small GTPase 1 (*RAC1*) signaling pathways [98,99,100].

#### 2.2.3. BCRT1

Polypyrimidine tract-binding protein 3 (*PTBP3*) can actuate the EMT process, invasive growth, and metastasis by increasing the stability of zinc finger E-box binding homeobox 1 (*ZEB1*) mRNA [101]. In breast cancer, BCRT1, a HIF-1α target gene, was identified to enhance PTBP3 expression via competitively binding with miR-1303 and promoting cell motility in vitro and lung metastasis in vivo [48] (Figure 1 and Table 1). Since PTBP3 can activate the translation of HIF-1α mRNA [102], a BCRT1/PTBP3/HIF-1α feedback loop may control cancer progression. Moreover, PTBP3 contributes to therapeutic resistance to gemcitabine under hypoxia [103], implying a possibility that BCRT1 regulates the sensitivity of cancer cells to therapeutic agents.

#### 2.2.4. FEZF1-AS1

FEZF1-AS1 is overexpressed and prompts cell proliferation, migration, invasion, and metastasis in different cancer types [104,105,106,107]. In pancreatic cancer, FEZF1-AS1 also expedites cell proliferation, migration, and invasion in vitro through interacting with miR-107 [108]. Furthermore, it was demonstrated that FEZF1-AS1 is increased by hypoxia, positively regulates HIF-1α expression via repressing the activity of miR-142-3p under hypoxia, and ultimately promotes cell invasion in pancreatic cancer [51] (Figure 1 and Table 1).

#### 2.2.5. H19 and HOTTIP

As stated in Section 2.1.1, H19 can upregulate BCL2, an anti-apoptotic factor, via blocking the activity of miR-612. Moreover, H19 was recognized to sponge miR-181d-5p, which directly targets β-catenin in glioblastoma [52]. Under hypoxic conditions, the knockdown of H19 lessens the expression of EMT markers, such as cadherin 2 (*CDH2*, also called N-cadherin) and snail family transcriptional repressor 1 (*SNAI1*), demonstrating a crucial role of H19 in the regulation of hypoxia-driven cell migration and invasion [52]. In this study, it was also confirmed that HIF-1α directly controls the transcription of H19 and SP1. Elevated SP1, in turn, stimulates H19 expression. These findings indicate that H19 expression is directly and indirectly regulated by HIF-1α [52] (Figure 1 and Table 1). Another study demonstrated that HOTTIP can increase the level of ZEB1 via sponging miR-101-3p, thereby promoting hypoxia-induced EMT in glioblastoma as well [60] (Figure 1 and Table 1). In line with this, miR-101-3p was suggested to repress EMT and metastasis in glioblastoma [109].

#### 2.2.6. HIFCAR

Screening of cancer-related lncRNAs identified that HIFCAR (also known as MIR31HG) is one of the hypoxia-responsive lncRNAs [56]. The migration and invasion of oral cancer cells are potentiated by HIFCAR. Furthermore, the downregulation of HIFCAR leads to the reduction of lung metastasis in vivo [56]. It was additionally found that the silencing of HIFCAR downregulates the level of HIF-1α target genes, including L1 cell adhesion molecule (*L1CAM*), without altering HIF-1α expression. Interestingly, it was delineated that HIFCAR physically interacts with HIF-1α, thereby recruiting HIF-1α to the promoter region of its target genes [56] (Figure 1 and Table 1).

#### 2.2.7. LINC01436 and NEAT1

In lung cancer, both LINC01436 and NEAT1 facilitate cell migration and invasion [65,68]. Hypoxia induces LINC01436 expression through downregulating E2F transcription factor 6 (E2F6), a transcription repressor of LINC01436. LINC01436 advances cancer growth and metastasis in vivo, and LINC01436 can exhibit its function by sponging miR-30a-3p that directly regulates HIF-2α (also known as endothelial PAS domain-containing protein 1 (*EPAS1*)) [65]. In the case of NEAT1, the transcription of this lncRNA is positively modulated by HIF-2α in lung cancer [68]. The knockdown of NEAT1 diminishes the effect of HIF-2α on cell migration, invasion, and the level of EMT markers [68], suggesting that NEAT1 facilitates EMT in a HIF-2α-dependent manner. A mechanism underlying NEAT1-mediated promotion of EMT indicated that miR-101-3p is inactivated by NEAT1, hence increasing the level of SRY-box transcription factor 9 (*SOX9*), an EMT- and Wnt/β-catenin signaling-activating factor [68] (Figure 1 and Table 1). Overall, these results also imply the feasibility that LINC01436 may elevate NEAT1 expression via the miR-30a-3p/HIF-2α axis.

#### 2.2.8. MAPKAPK5-AS1

MAPKAPK5-AS1 has been recognized as an oncogenic lncRNA [110,111,112]. MAPKAPK5-AS1 binds to enhancer of zeste homolog 2 (*EZH2*), leading to the transcriptional repression of cyclin-dependent kinase inhibitor 1A (*CDKN1A*, also known as *p21Cip1*). The downregulation of MAPKAPK5-AS1 induces cell cycle arrest and apoptotic cell death in colorectal cancer [110]. In addition, MAPKAPK5-AS1 can sponge let-7f-1-3p and cis-regulate the expression of MAPKAP kinase 5 (*MK5*), consequently upregulating SNAI1 to promote EMT [111]. Moreover, MAPKAPK5-AS1 advances the migration and invasion ability of thyroid cancer cells by constraining miR-519e-5p [112]. In hepatocellular carcinoma, MAPKAPK5-AS1 was confirmed as a HIF-1α-responsive lncRNA [67]. This lncRNA mediates the de-repression of PLAG1-like zinc finger 2 (*PLAGL2*), a miR-154-5p target, thus enhancing the EMT process and cell invasion in vitro and lung metastasis in vivo. PLAGL2 upregulated by MAPKAPK5-AS1 can successively increase HIF-1α, showing the presence of a HIF-1α-MAPKAPK5-AS1-PLAGL2 feedback loop [67] (Figure 1 and Table 1).

#### 2.2.9. NORAD and NUTF2P3-001

NORAD and NUTF2P3-001 are transcriptionally activated by hypoxia and serve as molecular sponges of miR-125a-3p and miR-3923, respectively, in pancreatic cancer [70,72]. In studies concerning them, miR-125a-3p and miR-3923 were proven to repress ras homolog family member A (*RHOA*) and Kirsten rat sarcoma viral oncogene homolog (*KRAS*), respectively. As a consequence, migration and invasion are prompted by these lncRNAs in vitro. It was also noticed that knockdown of either NORAD or NUTF2P3-001 significantly represses metastasis in vivo [70,72] (Figure 1 and Table 1). In another study, it was proposed that NORAD is downregulated in lung and breast cancer and that the overexpression of NORAD impedes metastasis in vivo [113]. These findings suggest that the function of NORAD is dissimilar depending on cancer types.

#### 2.2.10. RP11-390F4.3

A reporter gene assay identified RP11-390F4.3 as a HIF-1α-induced lncRNA [74]. The overexpression of RP11-390F4.3 facilitates in vitro cell migration/invasion together with an increase in EMT-related genes and potentiates in vivo metastatic activity of cancer cells [74] (Figure 1 and Table 1). Although the mechanism underlying oncogenic activities of this lncRNA is undisclosed, RP11-390F4.3 can be a feasible target for cancer treatments.

#### 2.2.11. UCA1

UCA1 has been discerned to limit apoptotic cell death and prompt migration, invasion, as well as metastasis by sponging diverse tumor-suppressive miRNAs, such as miR-143, miR-182-5p, and miR-203 [39,114,115]. Moreover, it was demonstrated that UCA1 is upregulated in hypoxia-resistant cancer cells generated by chronic hypoxia exposure, and that this lncRNA contributes to the augmentation of cell migration [75]. Additional evidence showed that UCA1 promotes cell migration due to its ability to inhibit miR-7-5p, thereby enhancing the level of epidermal growth factor receptor (*EGFR*) in hypoxia-resistant cancer cells [75] (Figure 1 and Table 1).

### 2.3. A lncRNA Controlling Angiogenesis

#### 2.3.1. RAB11B-AS1

In response to hypoxia, HIF-2α positively regulates the expression of RAB11B-AS1 in breast cancer [73]. RAB11B-AS1 can interact with RNA polymerase II (*POL II*) and enhance the recruitment of POL II to the promoters of pro-angiogenic genes, including VEGFA and angiopoietin-like 4 (*ANGPTL4*). Therefore, the overexpression of RAB11B-AS1 elevates these angiogenic factors, thereby favoring microvessel formation and distant metastasis in vivo [73] (Figure 1 and Table 1). By contrast, RAB11B-AS1 acts as a tumor suppressor through inhibiting proliferation, migration, invasiveness, and cell viability in osteosarcoma [116], implying a context-dependent role of RAB11B-AS1 in cancer.

#### 2.3.2. HITT

HIF-1α was found to degrade HITT via inducing miR-205 expression. Further, HITT represses the translation of HIF-1α [58]. These results suggest that HITT regulates HIF-1α expression at both transcription and post-transcription levels and that there is a regulatory loop between HIF-1α and HITT (also see Section 2.1.2). A mechanistic study demonstrated that HITT can directly bind to YBX1, a translational activator of HIF-1α, thus limiting the physical association between YBX1 and HIF-1α [58]. Functional evidence showed that the overexpression of HITT results in a decrease in VEGF levels and abates the growth of colorectal cancer in vivo [58] (Figure 1 and Table 1).

### 2.4. LncRNAs Related to Stemness and Drug Resistance

#### 2.4.1. HIF1A-AS2

HIF1A-AS2 can maintain stemness and confer resistance to cisplatin [54,55]. HIF1A-AS2 is abundant in mesenchymal glioma stem cells (M-GSCs) compared to proneural GSCs, indicating that HIF1A-AS2 is a lncRNA showing a subtype-specific expression pattern [54]. In this study, HIF1A-AS2 was supposed to stabilize high-mobility group AT-hook (*HMGA1*) at the mRNA level and increase its protein levels via interacting with RNA-binding proteins, namely DExH-box helicase 9 (*DHX9*) and insulin-like growth factor 2 mRNA-binding protein 2 (*IGF2BP2*) [54]. The depletion of HIF1A-AS2 leads to the reduction of cell viability and neurosphere-forming capacity of M-GSCs in vitro. Moreover, HIF1A-AS2 knockdown extends survival in intracranial xenograft models [54] (Figure 1 and Table 1). HMGA1 was demonstrated to support stemness at least partly by activating Notch signaling [117], suggesting that HIF1A-AS2 may activate Notch signaling via the DHX9/IGF2BP2/HMGA1 axis to maintain stemness.

Treatments with CoCl2, a hypoxia-mimetic agent, upregulate HIF1A-AS2 in bladder cancer cells. In addition, the expression of both HIF-1α and HIF1A-AS2 is upregulated in cisplatin-resistant bladder cancer cells (CRBC cells), denoting that HIF1A-AS2 can be regulated by HIF-1α in drug-resistant cells [55]. HIF1A-AS2 increases the level of HMGA1 in CRBC cells, consequently lowering the transcriptional activities of tumor suppressor P53 (*TP53*), TP63, and TP73, in addition to the level of BCL2-associated X protein (*BAX*). As expected, HIF1A-AS2 knockdown re-sensitizes CRBC cells to cisplatin via promoting apoptotic cell death [55]. Since DHX9 and IGF2BP2 are involved in HIF1A-AS2-mediated increase in HMGA1 expression as stated above, HIF1A-AS2 may regulate HMGA1 levels through physical interaction with DHX9 and IGF2BP2 in CRBC cells (Figure 1 and Table 1).

#### 2.4.2. KB-1980E6.3

IGF2BP1 can maintain stemness properties by stabilizing IGF2 mRNA and positively regulating the expression of aldehyde dehydrogenase 1 family member A1 (*ALDH1A1*) [118,119]. IGF2BP1 is also known to stabilize V-Myc avian myelocytomatosis viral oncogene homolog (*MYC*) mRNA, a stemness-promoting factor [120]. A recent study revealed that KB-1980E6.3 makes MYC mRNA more stable via recruiting IGF2BP1, thereby facilitating the self-renewal and in vivo tumorigenesis of breast cancer stem cells [62] (Figure 1 and Table 1). Since IGF2BP1 can regulate several stemness-related factors as mentioned above, further investigation into the function of KB-1980E6.3 is warranted.

### 2.5. LncRNAs and Glycolysis

#### 2.5.1. CASC9

Several studies defined CASC9 as an oncogenic factor due to its ability to facilitate tumorigenesis through activating TGF-β, extracellular signal-regulated kinase (*ERK*), and STAT3 signaling [121,122,123]. Additionally, CASC9 can bring about EGFR-mediated AKT activation by sponging miR-488-3p [124]. Further, it was connoted that CASC9, a hypoxia-inducible lncRNA, is regulated by HIF-1α and drives glycolysis via the upregulation of hexokinase 2 (*HK2*), lactate dehydrogenase A (*LDHA*), and glucose transporter type 4 (*GLUT4*) levels in pancreatic cancer [49]. Moreover, both AKT activation and HIF-1α induction are mediated by CASC9. Pharmacological inhibition of AKT diminishes glycolysis as well as HIF-1α levels, indicating a contribution of AKT to CASC9-induced glycolysis and HIF-1α regulation. These results also show a reciprocal regulation between CASC9 and HIF-1α. Moreover, the growth and metastasis of pancreatic cancer are impeded by silencing CASC9, suggesting that CASC9-induced glycolysis is responsible for pancreatic cancer malignancy [49] (Figure 1 and Table 1).

#### 2.5.2. HAND2-AS1

HAND2-AS1 is downregulated in various cancer types and negatively acts on cell proliferation, viability, migration/invasion, and metastasis [125,126,127]. In gastric cancer, it was demonstrated that the expression of both HAND2-AS1 and HIF-3α is downregulated by hypoxic conditions [53]. The overexpression of HAND2-AS1 impedes hypoxia-mediated cell migration, invasion, as well as glycolysis in gastric cancer cells. It was proposed that such tumor-suppressive effects of HAND2-AS1 can be due to the inhibitory action of HAND2-AS1 on miR-184, which targets HIF-3α [53] (Figure 1 and Table 1).

#### 2.5.3. HOTAIR and NPSR1-AS1

In hepatocellular carcinoma, both HOTAIR and NPSR1-AS1 are induced by hypoxia and can impel glycolysis under hypoxia [59,71]. HOTAIR serves as a decoy of miR-130a-3p that hinders glycolysis by targeting HIF-1α [59], indicating the role of HOTAIR as a positive feedback regulator of HIF-1α as well (Figure 1 and Table 1).

NPSR1-AS1 was found to activate ERK and elevate the level of pyruvate kinase M2 (*PKM2*), a glycolysis-promoting enzyme [71] (Figure 1 and Table 1). ERK can also mediate the nuclear translocation of PKM2, resulting in the transcriptional induction of glycolytic genes such as LDHA [128]. In additional studies, it was shown that ERK is able to induce Nima-related kinase 2 (*NEK2*) and that the expression of PKM2 can be positively regulated by NEK2 [129,130]. Therefore, NPSR1-AS1 may promote glycolysis via PKM2 nuclear translocation and the ERK/NEK2/PKM2 pathway.

#### 2.5.4. HOTTIP

HOTTIP was demonstrated to activate hypoxia-induced glycolysis in lung cancer [61]. In this study, it was supposed that HOTTIP absorbs miR-615-3p and increases glycolysis via upregulating the level of high-mobility group box 3 (*HMGB3*) [61] (Figure 1 and Table 1). Since HMGB3 was reported to activate ERK [131], it is feasible that glycolysis is enhanced via the HOTTIP/HMGB3/ERK axis. Further, a recent study revealed that ZEB1 can transcriptionally activate phosphofructokinase-M (*PFKM*), thus enhancing glycolysis [132]. Given HOTTIP’s role in ZEB1 regulation (Section 2.2.5), PFKM could be one of the mediators of HOTTIP-induced glycolysis.

#### 2.5.5. NEAT1

In anaplastic thyroid cancer, glycolysis can be repressed by NEAT1 silencing under hypoxia. Additionally, in vivo growth of thyroid cancer is retarded by the depletion of NEAT1 [69]. In this study, it was further demonstrated that NEAT1 sponges both miR-206 and miR-599. The knockdown of either miR-206 or miR-599 increases lactate production and HK2 levels in NEAT1-silencing cells, indicating their involvement in the regulation of signaling pathways related to glycolysis [69] (Figure 1 and Table 1). In addition, NEAT1 may positively regulate glycolysis via Wnt/β-catenin signaling, which can enhance glycolysis through multiple downstream factors such as AKT [133] (also see Section 2.2.7 about NEAT1 and Wnt/β-catenin).

#### 2.5.6. XIST

XIST elevates cell motility and glycolysis in vitro via confining the activity of miR-381-3p, which directly targets NEK5. In addition, XIST enhances in vivo growth of nasopharyngeal carcinoma [76]. Although the mechanism by which NEK5 regulates glycolysis remains obscure, the knockdown of NEK5 was found to suppress hypoxia-induced glycolysis [76] (Figure 1 and Table 1). Since NEK5 can increase the expression of mitochondrial ATP-dependent protease Lon (*LONP1*) [134], which is able to serve as a glycolysis-enhancing factor [135], the miR-381-3p/NEK5/LONP1 axis may be involved in XIST-induced glycolysis.

## 3. LncRNAs Regulating HIF-1α Expression

As was the case in Section 2, we classified HIF-1α-regulating lncRNAs into six categories depending on what lncRNAs are involved in cellular events, aiming to display the function of each lncRNA even though they can have multiple functions.

### 3.1. LncRNAs Affecting Cell Survival and Apoptosis

#### 3.1.1. CDKN2B-AS1

**Figure 2:**
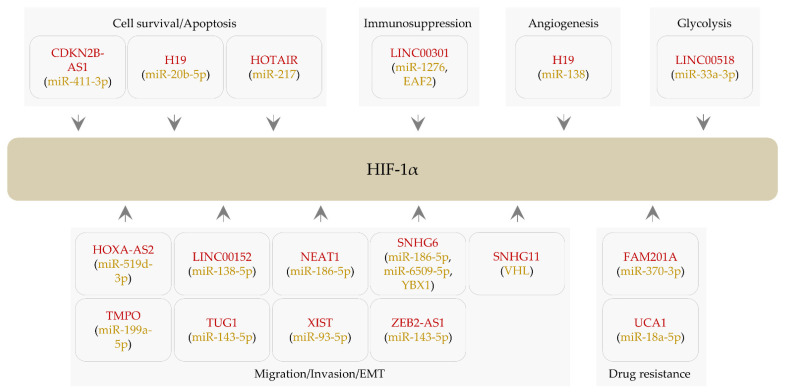
LncRNAs modulating the level of HIF-1α. Rounded rectangles represent lncRNAs (red). Rounded brackets denote cellular factors (proteins and miRNAs) involved in lncRNA-mediated HIF-1α regulation. Positive regulation is shown by an arrow.

It has been demonstrated that CDKN2B-AS1 is highly expressed in various cancer types and serves as an oncogenic factor by regulating multiple cellular events such as apoptosis [136,137]. Further evidence showed that CDKN2B-AS1 interacts with miR-411–3p, which directly targets HIF-1α in ovarian cancer [138]. The knockdown of CDKN2B-AS1 induces caspase-3 activation and apoptotic cell death via reducing HIF-1α expression and p38 activity. In addition, the in vivo growth of ovarian cancer cells is hampered by CDKN2B-AS1 depletion [138] (Figure 2 and Table 2). Hypoxia is known to activate p38, thus leading to cancer aggressiveness via enhancing cell survival [139,140]. Moreover, HIF-1α is activated by p38 [139]. These results imply that both expression and activity of HIF-1α can be positively regulated by CDKN2B-AS1.

#### 3.1.2. H19 and HOTAIR

AXL receptor tyrosine kinase (*AXL*) stimulates pro-survival signaling to protect cells from apoptosis, and its expression can be transcriptionally activated by HIF-1 and HIF-2 [159,160,161]. Recent studies demonstrated that both H19 and HOTAIR facilitate AXL expression, thereby inhibiting apoptosis induction in vitro [142,144]. It was also noted that the knockdown of H19 and HOTAIR retards the growth of endometrial cancer and renal cell carcinoma in vivo, respectively. Mechanistically, H19 and HOTAIR antagonize miR-20b-5p and miR-217, respectively, thus enhancing the expression of HIF-1α and AXL [142,144] (Figure 2 and Table 2).

### 3.2. LncRNAs Regulating Cell Migration, Invasion, and EMT

#### 3.2.1. HOXA-AS2

It has been shown that miR-519d-3p negatively controls cell proliferation, migration, and invasion by, for example, restraining Wnt/β-catenin, p38, and PI3K/AKT signaling [162,163,164]. HOXA-AS2 was noticed to inactivate miR-519d-3p, thus reinforcing the migration and invasion of nasopharyngeal carcinoma cells. In a study concerning them, miR-519d-3p was confirmed to target HIF-1α [145] (Figure 2 and Table 2). Another study has shown the direct restraint of HIF-2α expression by miR-519d-3p [165]. These data imply the possibility of modulation of the hypoxia signaling pathway via the HOXA-AS2/miR-519d-3p axis and the feasibility of targeting HOXA-AS2 for cancer therapy.

#### 3.2.2. LINC00152

In multiple cancers, LINC00152 supports EMT and metastasis by positively regulating the level of ZEB1, PI3K, and AKT [166,167]. In gallbladder cancer, LINC00152 was also observed to exhibit EMT- and metastasis-promoting activities via sponging miR-138-5p that targets HIF-1α [146] (Figure 2 and Table 2). The transcription of LINC00152 is activated by krueppel-like factor 5 (*KLF5*) [168], and KLF5 levels can be increased by hypoxia [169]. Therefore, the existence of a hypoxia/ KLF5/LINC00152/HIF-1α signaling loop is worth considering.

#### 3.2.3. NEAT1 and TUG1

In addition to being controlled by hypoxia (Section 2.2.7, Section 2.5.5, and Table 1), NEAT1 can lead to a rise in HIF-1α levels via deactivating miR-186-5p [149] (Figure 2 and Table 2). The overexpression of NEAT1 provokes EMT, whereas EMT is abrogated by NEAT1 silencing in osteosarcoma cells. Additionally, the in vivo growth of osteosarcoma was noticed to be significantly hampered by NEAT1 silencing [149].

TUG1 also boosts the level of HIF-1α by sponging miR-143-5p, thus driving the invasion, peritoneal spreading, and metastasis of osteosarcoma [155] (Figure 2 and Table 2). In this study, it was additionally discovered that TGF-β derived from CAFs can increase the expression of TUG1 in osteosarcoma cells, indicating the contribution of TUG1 to CAF-mediated control of osteosarcoma progression [155].

Overall, these findings suggest that NEAT1 and TUG1 are attractive targets for osteosarcoma therapy.

#### 3.2.4. SNHG6

Numerous studies have shown that cancer progression is fostered by SNHG6 [170,171,172,173]. Moreover, SNHG6 can elevate the expression of HIF-1α by either sponging miRNAs or enhancing the translation of HIF-1α mRNA [150,151,152].

SNHG6 was confirmed to stimulate the migration and invasion of esophageal cancer cells by absorbing miR-186-5p, which directly targets HIF-1α [150] (Figure 2 and Table 2).

Moreover, SNHG6 subdues the activity of miR-6509-5p. As a consequence, SNHG6 enhances migration and invasion abilities of hepatocellular carcinoma cells, along with an increase in HIF-1α expression. In xenografts, the growth of hepatocellular carcinoma is suppressed by the downregulation of SNHG6 [151] (Figure 2 and Table 2).

Furthermore, the pro-tumorigenic effect of SNHG6 was also reported in clear cell renal cell carcinoma [152]. In a study concerning them, it was proposed that SNHG6 interacts with Y-box binding protein 1 (*YBX1*, also called *YB1*) and mediates the connection between YBX1 proteins and HIF-1α mRNAs to activate translation of HIF-1α transcripts [152] (Figure 2 and Table 2).

#### 3.2.5. SNHG11 and XIST

Von Hippel-Lindau tumor suppressor (*VHL*) can bind to and degrade HIF-1α via the ubiquitin–proteasome pathway [174,175]. A recent publication described that SNHG11 physically interacts with and stabilizes HIF-1α proteins by blocking the binding of HIF-1α to VHL. Consequently, SNHG11 facilitates hypoxia-induced migration and invasion in vitro and the lung metastasis of colorectal cancer cells in vivo [153] (Figure 2 and Table 2). It is also acknowledged that SNHG11 upregulates MYC expression [176]. Since MYC can post-transcriptionally stabilize HIF-1α [177], SNHG11 may regulate the stability of HIF-1α, at least partly via VHL and MYC.

In colorectal cancer, XIST also augments the level of HIF-1α via negatively regulating miR-93-5p activity; therefore, XIST can possess stimulatory effects on migration, invasion, and the EMT process. Further, the overexpression and downregulation of XIST led to an increase and a decrease in the growth of colorectal cancer, respectively, in a xenograft model [157] (Figure 2 and Table 2). Since XIST positively controls MYC expression via Wnt/β-catenin signaling [178], it is feasible that XIST may post-transcriptionally stabilize HIF-1α as well.

#### 3.2.6. TMPO-AS1

Accumulating evidence shows that TMPO-AS1 exerts oncogenic functions in diverse cancer types. For instance, TMPO-AS1 and miR-383-5p act competitively in their interaction with SOX11, which can accelerate the migration and invasion of pancreatic cancer cells. As a result, the downregulation of TMPO-AS1 restrains cell migration and invasion in vitro and the growth of pancreatic cancer cells in vivo [179]. In addition, TMPO-AS1 can accelerate cancer progression via activating AKT/mechanistic target of rapamycin kinase (mTOR) signaling [180,181]. Furthermore, the malignant phenotype of retinoblastoma cells is fueled by TMPO-AS1, owing to its ability to inhibit miR-199a-5p, which targets HIF-1α [154] (Figure 2 and Table 2).

#### 3.2.7. ZEB2-AS1

In gastric cancer, ZEB2-AS1 can heighten the level of HIF-1α by obstructing the activity of miR-143-5p, provoking the invasion of gastric cancer cells. As expected, the depletion of ZEB2-AS1 significantly hinders the growth of gastric cancer in vivo [158] (Figure 2 and Table 2). ZEB2-AS1 was found to escalate the level of zinc finger E-box-binding homeobox 2 (*ZEB2*), thus promoting EMT and metastasis [182,183]. In another study, ZEB2-AS1 was confirmed to activate Wnt/β-catenin signaling via augmenting ZEB2 expression, hence showing a growth-promoting effect in gastric cancer in vivo [184]. Therefore, HIF-1α can also be stabilized by the ZEB2-AS1/Wnt/β-catenin axis (see Section 3.2.5 about the relationship between HIF-1α and Wnt/β-catenin).

### 3.3. LncRNAs Modulating Angiogenesis

#### H19

As stated in Section 2.1.1, Section 2.2.5 and Section 3.1.2, H19 has a cell survival- and EMT-promoting activity. Further, H19 can trigger angiogenesis by regulating several factors. In glioma, H19 increases vasohibin 2 (*VASH2*) levels and Wnt/β-catenin signaling via impairing the action of miR-29a-3p and miR-342, respectively, actuating angiogenesis as a consequence [185,186]. By inhibiting miR-29b-3p activities, H19 also activates angiogenesis as well as metastasis in bladder cancer [187]. Further, recent mechanistic evidence showed that H19 upregulates the expression of VEGF by interfering with miR-138, which targets HIF-1α [143] (Figure 2 and Table 2).

### 3.4. LncRNAs Affecting Drug Resistance

#### 3.4.1. FAM201A

EGFR is commonly overexpressed in cancer and renders cells resistant to radiotherapy [188,189,190]. EGFR inhibition has been shown to sensitize cancer cells to radiation therapy through potentiating, for instance, cell cycle arrest and apoptosis [191]. HIF-1α also promotes radioresistance by regulating multiple cellular events, such as mitochondrial biogenesis, apoptosis, and EMT [192,193,194]. FAM201A was recently proven to modulate the effect of radiotherapy in lung cancer [141]. The silencing of FAM201A significantly reduces cell proliferation together with an induction of apoptosis in irradiated cells. The efficacy of irradiation is also improved by FAM201A knockdown in lung cancer xenografts. Such radioresistant-promoting effects of FAM201A could be due to its sequestering property towards miR-370-3p, which targets EGFR and HIF-1α [141] (Figure 2 and Table 2).

#### 3.4.2. UCA1

Evidence from an in vitro study suggested that UCA1 silencing inactivates AKT and mTOR, augmenting tamoxifen-induced apoptosis in breast cancer cells [195]. Similarly, it was denoted that ectopic expression of UCA1 desensitizes breast cancer cells to tamoxifen along with an insufficient activation of caspase-3 [156]. It was found that treatments with tamoxifen caused the induction of HIF-1α and UCA1 expression. UCA1 was validated to sponge miR-18a-5p that directly represses HIF-1α (Figure 2 and Table 2). Furthermore, it was shown that tamoxifen-induced UCA1 is abrogated by HIF-1α silencing, illustrating a feedback loop between UCA1 and HIF-1α [156].

### 3.5. A lncRNA and Immunosupression

#### LINC00301

Recently, LINC00301 was demonstrated to be responsible for the creation of an immunosuppressive microenvironment in lung cancer [147]. LINC00301 sponges miR-1276 to upregulate HIF-1α expression. In addition, LINC00301 is able to augment HIF-1α levels by transcriptionally repressing the expression of ELL-associated factor 2 (*EAF2*), which is known to stabilize VHL (see Section 3.2.5 about VHL and HIF-1α). Thus, LINC00301 can increase the number of tumor-infiltrating Tregs in vivo. It was also observed that transcriptional activation of LINC00301 is mediated by Forkhead box C1 (*FOXC1*) [147] (Figure 2 and Table 2). Considering that FOXC1 is induced by HIF-1α under hypoxia [196], the existence of a LINC00301-HIF-1α-FOXC1 feedback loop is feasible.

### 3.6. LncRNAs and Glycolysis

#### LINC00518

LINC00518 is potentially involved in cancer-related processes, such as cell viability, migration, invasion, and metastasis [197,198,199,200]. Furthermore, LINC00518 can promote therapeutic resistance to various agents, including paclitaxel, vincristine, and adriamycin [201,202]. Moreover, LINC00518 was determined to promote HIF-1α expression by targeting miR-33a-3p in melanoma cells, consequently inducing glycolysis-mediated radioresistance in vitro and in vivo [148] (Figure 2 and Table 2).

## 4. Conclusions

Since hypoxia broadly impacts molecular events involved in cancer progression, aggressiveness, and therapeutic resistance, targeting hypoxia is an attractive approach in the management of solid cancers [1,203]. To surmount and exploit this distinctive feature of solid cancer, efforts to develop HIF inhibitors and hypoxia-activated prodrugs have been ongoing for targeting oncogenic signaling pathways mediated by hypoxia and HIFs [203,204]. For this strategy, further studies are still desired to overcome limiting factors such as dose-limiting toxicity. In addition, the development of resistance is unavoidable. For instance, prolonged exposure to PT2399, a selective HIF-2 inhibitor, leads to the development of resistance that is associated with an increase in tumor vascularity and VEGF levels [205]. Thus, new treatment strategies are necessary to refine therapeutic benefits.

**Figure 3:**
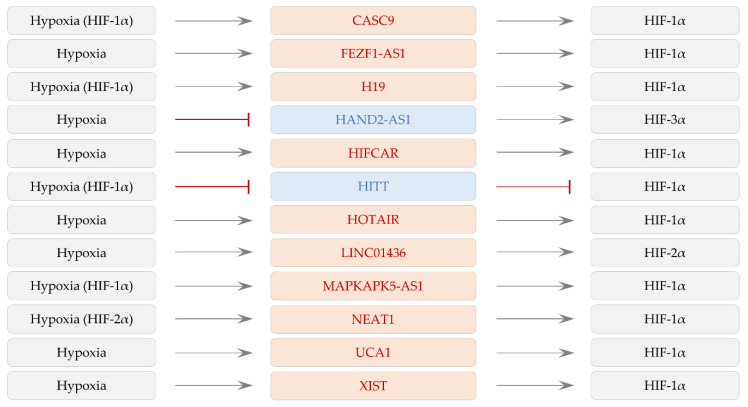
The regulatory loop between lncRNAs and hypoxia/HIFs. Red rounded rectangles present oncogenic lncRNAs. Tumor-suppressive lncRNAs are shown in blue rounded rectangles. At least through HIF-1α or HIF-2α (displayed in parentheses), hypoxia can modulate the levels of lncRNAs, which in turn affect the expression and activity of HIF-α. The absence of parentheses next to hypoxia suggests the regulation of lncRNAs, possibly in HIF-dependent and/or -independent manners under hypoxia, and that further studies are necessary. Positive regulation is shown by an arrow. An inhibitory effect is designated by a perpendicular line.

Accumulating evidence described here shows that the levels of lncRNAs can be affected by hypoxia/HIFs and that lncRNAs control the expression and activity of HIF-α subunits. Among lncRNAs in Section 2 and Section 3, some lncRNAs can form a regulatory feedback loop with hypoxia/HIF subunits as shown in Figure 3. Although experimental confirmation is needed, other lncRNAs may also regulate hypoxia signaling via creating a feedback loop with HIF-1α (Section 2.2.3, Section 3.2.2 and Section 3.5) and reinforcing the level of both HIF-1α and HIF-2α (Section 3.2.1). Under hypoxia, HIFs can directly induce lncRNAs. Additionally, HIFs may control the level and activity of other transcription factors, indirectly altering lncRNA levels. Moreover, the expression of lncRNAs can be upregulated or downregulated in a HIF-independent manner. Additionally, the cytoplasmic localization of LINC00152 is stimulated by hypoxia [206], suggesting that hypoxia can modulate the function of lncRNAs not only by altering their expression but also by controlling their intracellular localization. More experimental approaches are necessary to analyze the profound relationship between hypoxia/HIFs and lncRNAs. Nonetheless, it suggests that lncRNA-based cancer therapy can be a potential strategy against cancers.

Growing evidence suggests that the modulation of lncRNA expression sensitizes cancer cells to anti-cancer agents [17,207,208]. Since a therapeutic response can be improved by combination therapy, exploring a novel strategy of lncRNA-based cancer therapy in combination with other hypoxia-targeting agents (e.g., HIF inhibitors and prodrugs) is worth considering. Moreover, extracellular vesicles (EVs) derived from cancer cells transport cargo molecules, such as lncRNAs, to other adjacent cells, eventually affecting cancer progression [209]. It has been reported that lncRNAs are incorporated in hypoxic cancer-cell-originated EVs. Examples include UCA1 and lincRNA-p21, both of which are delivered to endothelial cells and promote angiogenesis [210,211]. Therefore, the combination of hypoxia-targeting agents with EV inhibitors can more effectively control cancers.

As mentioned in Section 2.2.9 and Section 2.3.1, lncRNAs can behave differently depending on cancer types. In addition, HIF1A-AS1 is overexpressed in hepatocellular carcinoma and supports cell survival [212], whereas this lncRNA was reported to promote apoptotic cell death induced by tumor necrosis factor-α in Kupffer cells [213], suggesting a possibility of context-specific functions of other lncRNAs. Further, both LINC00511 and miR-31-5p are oncogenic noncoding RNAs in colorectal cancer [214] (see Section 2.1.4 about LINC00511). However, a recent study demonstrated that LINC00511 can sponge miR-31-5p [215], implying intricate lncRNA–miRNA networks. To establish a promising strategy for lncRNA-based cancer therapy, it is crucial to attentively consider these features of lncRNAs.

LncRNAs can regulate a broad range of cellular signaling regardless of oxygen levels [17,216,217], and solid cancers are heterogeneous in terms of oxygenation [218]. Therefore, targeting an individual lncRNA can have a chance of controlling both well-oxygenated and hypoxic cancer cells. Advanced knowledge of lncRNAs will enable lncRNA-based cancer therapy to progress toward clinical application.

## Figures and Tables

**Table 1:** The list of lncRNAs that are modulated by hypoxia and HIFs (alphabetical order).

LncRNA	Type of Cancer	Expression (Cell Lines and/or Tissues)	Induction Condition	In Vivo Experiment	Clinical Relevance	Ref.
AC093818.1	Breast cancer	Overexpressed in triple-negative breast cancer tissues and cell lines (BT-20, MDA-MB-231, MDA-MB-468, and SUM159)	Upregulated in MDA-MB-231 and SUM159 cells by hypoxia (1% O_2_)	Orthotopic implantation of MDA-MB-231 cells stably knocking down AC093818.1	–	[46]
AGAP2-AS1	Hepatocellular carcinoma	Abundantly expressed in cancer tissues and cell lines (Hep3B, SMCC-7721, Huh7, HCCLM3, and MHCC-97H)	Increased in Hep3B cells under hypoxia	Tail vein injections of AGAP2-AS1-overexpressing Hep3B cells or AGAP2-AS1-silencing HCCLM3 cells	Poor overall survival of patients with high AGAP2-AS1 expression	[47]
BCRT1	Breast cancer	Upregulated in cancer tissues compared to normal controls	Increased in MDA-MB-231 and MDA-MB-468 cells under hypoxic stress	Subcutaneous or tail vein injections of MDA-MB-231 cells stably overexpressing BCRT1	High expression of BCRT1 is correlated with poor overall survival and disease-free survival	[48]
CASC9	Pancreatic cancer	–	Increased in PANC-1 and SW1990 cells by hypoxia (1% O_2_)	Subcutaneous or tail vein injections of CASC9-depleted SW1990 cells		[49]
EIF3J-AS1	Hepatocellular carcinoma	Upregulated in cancer tissues and cell lines (HepG2, SMCC-7721, HCCLM3, and MHCC-97H)	Induced by hypoxia in SMCC-7721 cells	–	Prognostic features (size, invasion and stages) are associated with EIF3J-AS1 levels	[50]
FEZF1-AS1	Pancreatic cancer	Upregulated in cancer tissues and cell lines (PANC-1, SW1990, HuP, and CFPAC-1)	Induced by hypoxia (1% O_2_) in PANC-1 and SW1990 cells	–	Positively associated with advanced TNM stages	[51]
H19	Cholangiocarcinoma	Upregulated in carcinoma tissues compared to normal bile duct tissues	Increased by HIF-1α overexpression	Subcutaneous injections of cholangiocarcinoma cells transduced with lentiviral vectors encoding small hairpin RNA (shRNA) against HIF-1α	–	[45]
	Glioblastoma	–	Increased in U87 and U251 cells following exposure to hypoxia (2% O_2_)	Subcutaneous injections of U87 cells stably knocking down HIF-1α	Patients with high H19 levels show poor overall survival	[52]
HAND2-AS1	Gastric cancer	Downregulated in cancer tissues compared to adjacent control tissues	Reduced by hypoxia (1% O_2_) in AGS cells	–	–	[53]
HIF1A-AS2	Glioblastoma multiforme	Abundantly expressed in cancer tissues	Upregulated in mesenchymal glioblastoma stem cells exposed to hypoxic conditions (1% O_2_)	Intracranial xenografts generated by implanting HIF1A-AS2-depleted mesenchymal glioblastoma stem cells	–	[54]
	Bladder cancer	Increased in cancer tissues from patients treated with cisplatin	Upregulated in cisplatin-resistant and cobalt chloride (CoCl2)-treated cells	–	–	[55]
HIFCAR	Oral cancer	Overexpressed in cancer tissues compared to non-cancerous tissues	Induced by hypoxia (1% O_2_) and CoCl2 treatment in HeLa cells	Tail vein injections of HIFCAR-depleted SAS cells	High HIFCAR levels are associated with worse overall survival, tumor differentiation, and lymph node metastasis	[56]
HITT	Colorectal cancer	Downregulated in cancer tissues compared to normal controls	Decreased by hypoxia (1% O_2_) in HCT116 and HeLa cells	Subcutaneous injections of HCT116 cells stably overexpressing HITT	Negatively associated with TNM classification	[57,58]
HOTAIR	Hepatocellular carcinoma	Upregulated in cancer tissues	Augmented in HepG2 and Huh7 cells after hypoxic exposure (1% O_2_)	–	–	[59]
HOTTIP	Glioblastoma	Upregulated in metastatic glioma tissues compared to non-metastatic tissues	Increased in U87 and U251 cells under hypoxia (1% O_2_)	–	Negatively correlated with the survival rate of patients	[60]
	Lung cancer	Abundant in cancer tissues compared to normal controls	Induced in A549 and H1299 cells following hypoxic exposure (1% O_2_)	–	–	[61]
KB-1980E6.3	Breast cancer	Highly expressed in cancer tissues compared to adjacent normal tissues	Elevated in multiple cell lines (e.g., BT549 and Hs578T) under hypoxic conditions (1% O_2_)	Subcutaneous injections of stem cells from Hs578T in which KB-1980E6.3 is silenced	Negatively correlated with the overall survival of patients	[62]
LINC00475	Glioblastoma	–	Upregulated in LN229 cells exposed to hypoxia (1% O_2_)	Injections of lentiviral vectors encoding shRNA against LINC00475 into mice bearing LN229 cells	High expression is correlated with the stage of cancer	[63]
LINC00511	Colorectal cancer	Abundantly expressed in cancer tissues compared to normal tissues	Transcription is promoted by HIF-1α overexpression	–	The level of LINC00511 is negatively correlated with the overall survival of patients	[64]
LINC01436	Lung cancer	Overexpressed in cancer tissues compared to adjacent normal tissues	Increased in H1299 cells under hypoxic conditions (1% O_2_)	Subcutaneous or tail vein injections of A549 cells stably overexpressing LINC01436	High levels are associated with worse overall survival of patients	[65]
MALAT1	Hepatocellular carcinoma	–	Increased in several cell lines (Huh7, SNU-423, PLC, and Hep3B) under hypoxic conditions	–	–	[66]
MAPKAPK5-AS1	Hepatocellular carcinoma	Highly expressed in cancer tissues	Increased by hypoxia (1% O_2_) in Hep3B cells	Subcutaneous or tail vein injections of MAPKAPK5-AS1-knockdown HCCLM3 cells or MAPKAPK5-AS1-overexpressing Hep3B cells	Positively associated with poor prognosis and pathological stages	[67]
NEAT1	Lung cancer	Abundantly expressed in cancer tissues	Upregulated by hypoxia (1% O_2_) in A549 and SPCA1 cells	–	Positively associated with the tumor, node and metastasis (TNM) classification	[68]
	Anaplastic thyroid cancer	Upregulated in cancer tissues	Increased in several cell lines (SW1736 and KAT-18) under hypoxic conditions (1% O_2_)	Subcutaneous injections of SW1736 cells stably knocking down NEAT1	–	[69]
NORAD	Pancreatic cancer	Upregulated in cancer tissues	Increased in SW1990 cells under hypoxia (1% O_2_)	Orthotopic implantation of SW1990 cells stably knocking down NORAD	Poor overall and recurrence-free survival in patients with high NORAD levels	[70]
NPSR1-AS1	Hepatocellular carcinoma	Overexpressed in cancer tissues compared to control specimens	Increased in Hep3B and Huh7 cells by hypoxia and HIF-1α	–	–	[71]
NUTF2P3-001	Pancreatic cancer	Overexpressed in cancer tissues compared to noncancerous tissues	Increased in hypoxia (1% O_2_)-exposed and CoCl2-treated PANC-1 cells	Subcutaneous injections of NUTF2P3-001-depleted PANC-1 cells	Strong expression is correlated with distant metastasis and worse prognosis	[72]
RAB11B-AS1	Breast cancer	Upregulated in cancer tissues	Induced by hypoxia (1% O_2_) in multiple cell lines (e.g., MDA-MB-231 and BT474)	Orthotopic implantation of MDA-MB-231 cells stably knocking down RAB11B-AS1	–	[73]
RP11-390F4.3	Multiple types (hypopharyngeal, breast, osteosarcoma, prostate, and lung cancer)	–	Induced by hypoxia (1% O_2_) in FADU, MCF-7, and U2-OS cells. Decreased by HIF-1α silencing in H1299, MDA-MB-231, and PC3 cells	Tail vein or orthotopic injections of FADU cells (RP11-390F4.3 overexpressed) and H1299/MDA-MB-231 cells (RP11-390F4.3 depleted)	–	[74]
UCA1	Gastric cancer	–	Increased in hypoxia-resistant cell lines (MGC-803 and BGC-823 cells)	–	–	[75]
XIST	Nasopharyngeal cancer	Overexpressed in cancer tissues	Increased by hypoxia (1% O_2_) in HK-1 and C666-1 cells	Subcutaneous injections of XIST-depleted HK-1 cells	–	[76]

**Table 2:** LncRNAs that regulate the expression of HIF-1α (alphabetical order).

LncRNA	Type of Cancer	Expression (Cell Lines and/or Tissues)	In Vivo Experiment	Clinical Relevance	Ref.
CDKN2B-AS1	Ovarian cancer	Highly expressed in cancer cells (e.g., SKOV-3 cells) compared to normal ovarian epithelial cells	Subcutaneous injections of SKOV-3 cells following the knockdown of CDKN2B-AS1	–	[138]
FAM201A	Lung cancer	Highly expressed in cancer tissues from patients responding poorly to radiotherapy	Subcutaneous injections of A549 and SK-MES-1 cells following FAM201A silencing	Unfavorable prognosis in patients with high FAM201A levels	[141]
H19	Endometrial cancer	Overexpressed in cancer tissues compared to normal controls	Subcutaneous injections of H19-silencing HHUA cells	–	[142]
	Glioblastoma	Abundant in cancer cell lines (U373, A172, and U87) compared to normal glial cells (HEB)	–	–	[143]
HOTAIR	Renal cancer	Upregulated in cancer tissues and cell lines compared to adjacent normal tissues and normal renal cells, respectively	Subcutaneous injections of 769-P cells transfected with HOTAIR small interfering RNA	High expression of HOTAIR is correlated with tumor stages and metastasis	[144]
HOXA-AS2	Nasopharyngeal cancer	Highly expressed in cancer tissues as well as cell lines (SUNE1 and SUNE2)	–	–	[145]
LINC00152	Gallbladder cancer	Abundant in cancer tissues and cell lines (NOZ and GBC-SD)	Intraperitoneal injections of GBC-SD cells stably overexpressing LINC00152	Positively associated with short overall survival and lymph node invasion	[146]
LINC00301	Lung cancer	Upregulated in cancer tissues compared to normal counterparts	Implantations of LINC00301-overexpressing LA-4 and KLN-205 cells	Positively associated with advanced clinical stage, lymph node metastasis, and worse overall survival	[147]
LINC00518	Melanoma	Overexpressed in cancer tissues compared to normal skin controls	Subcutaneous injections of LINC00518-depleted WM451 and A375 cells + irradiation (2Gy)	Worse survival in patients with high LINC00518 levels	[148]
NEAT1	Osteosarcoma	Enriched in cancer tissues and various cell lines (HOS, U2OS, SaOS2, and MG63)	Subcutaneous injections of HOS cells following NEAT1 depletion	Significantly associated with distant metastasis, advanced clinical stage, and poor overall survival	[149]
SNHG6	Esophageal cancer	Upregulated in cancer tissues and cell lines (EC109, EC9706, KYSE30, and KYSE150)	–	–	[150]
	Hepatocellular carcinoma	Increased in cancer tissues compared to control tissues	Subcutaneous injections of Huh7 cells stably knocking down SNHG6	Associated with overall and progression-free survival	[151]
	Clear cell renal cell carcinoma	Highly expressed in cancer tissues compared to normal tissues	Subcutaneous injections of A498 cells stably expressing SNHG6	Short overall survival in patients with high SNHG6 levels	[152]
SNHG11	Colorectal cancer	Highly expressed in cancer tissues compared to normal tissues	Tail vein injections of HCT116 cells stably overexpressing SNHG11	Positively associated with lymphatic invasion, metastasis, distant recurrence, and short overall survival	[153]
TMPO-AS1	Retinoblastoma	Overexpressed in cancer tissues compared to adjacent normal tissues	–	Positively associated with the stages of cancer	[154]
TUG1	Osteosarcoma	Highly expressed in cancer tissues compared to normal controls. Higher in several cancer cell lines (e.g., U2OS and 143B cells) than in NHOst (normal osteoplastic cells)	Subcutaneous, intraperitoneal, or intravenous injections of TUG1-depleted U2OS cells	Positively associated with poor prognosis	[155]
UCA1	Breast cancer	Abundant in tamoxifen-resistant cell lines (LCC2, LCC9, and BT474) compared to a tamoxifen-sensitive cell line (MCF-7)	–	–	[156]
XIST	Colorectal cancer	Upregulated in cancer tissues compared to normal controls	Subcutaneously inject XIST-silencing LoVo cells or SW480 cells overexpressing XIST	Positively associated with the TNM stage	[157]
ZEB2-AS1	Gastric cancer	Overexpressed in cancer cell lines (SGC-7901, BGC-823, and MKN-28) compared to normal gastric epithelial cells (GES-1)	Subcutaneous injections of SGC-7901 cells depleted of ZEB2-AS1	–	[158]

## Abbreviations

3′ UTR	3′ untranslated region
AGAP2	ArfGAP with GTPase domain, ankyrin repeat and PH domain 2
AKT2	AKT serine/threonine kinase 2
ALDH1A1	Aldehyde dehydrogenase 1 family member A1
ANGPTL4	Angiopoietin-like 4
ANXA11	Annexin A11
AXL	AXL receptor tyrosine kinase
BAX	BCL2-associated X protein
BCL2	B-cell CLL/lymphoma 2
BID	BH3-interacting domain death agonist
BRD4	Bromodomain-containing protein 4
CAFs	Cancer-associated fibroblasts
CCL28	C-C motif chemokine ligand 28
CDH2	Cadherin 2
CDKN1A	Cyclin-dependent kinase inhibitor 1A
CoCl2	Cobalt chloride
CREPT	Cell-cycle related and expression-elevated protein in tumor
CTNND2	Catenin delta 2
DHX9	DExH-box helicase 9
EAF2	ELL-associated factor 2
EGFR	Epidermal growth factor receptor
EMT	Epithelial-to-mesenchymal transition
EPAS1	Endothelial PAS domain-containing protein 1
ERK	Extracellular signal-regulated kinase
EVs	Extracellular vesicles
EZH2	Enhancer of zeste homolog 2
FOXC1	Forkhead box C1
GLUT4	Glucose transporter type 4
HIFs	Hypoxia-inducible factors
HK2	Hexokinase 2
HMGA1	High-mobility group AT-hook
HMGB3	High-mobility group box 3
IGF2BP2	Insulin-like growth factor 2 mRNA-binding protein 2
ITGA6	Integrin subunit alpha 6
KLF5	Krueppel-like factor 5
KRAS	Kirsten rat sarcoma viral oncogene homolog
L1CAM	L1 cell adhesion molecule
LDHA	Lactate dehydrogenase A
LncRNAs	Long noncoding RNAs
LONP1	Mitochondrial ATP-dependent protease Lon
MCL1	Myeloid cell leukemia 1
M-GSCs	Mesenchymal glioma stem cells
miRNAs	MicroRNAs
MK5	MAPKAP kinase 5
mRNAs	Messenger RNAs
mTOR	Mechanistic target of rapamycin kinase
MYC	V-Myc avian myelocytomatosis viral oncogene homolog
NEK2	Nima-related kinase 2
NFIA	Nuclear factor I/A
NFYA	Nuclear transcription factor Y subunit alpha
NOB1	NIN1/PSMD8 binding protein 1 homolog
PDK1	Pyruvate dehydrogenase kinase 1
PFKM	Phosphofructokinase-M
PIKE	Phosphatidylinositol 3-kinase enhancer
PKM2	Pyruvate kinase M2
PLAGL2	PLAG1-like zinc finger 2
POL II	RNA polymerase II
PRC2	Polycomb repressive complex 2
PTBP3	Polypyrimidine tract-binding protein 3
RAC1	Rac family small GTPase 1
RHOA	Ras homolog family member A
shRNA	Small hairpin RNA
SNAI1	Snail family transcriptional repressor 1
SP1	Sp1 transcription Factor
STAT3	Signal transducer and activator of transcription 3
TGF-β	Transforming growth factor β
TNM	Tumor, node and metastasis
TP53	Tumor suppressor P53
Tregs	Regulatory T cells
VASH2	Vasohibin 2
VEGF	Vascular endothelial growth factor
VHL	Von Hippel-Lindau tumor suppressor
WNT2B	Wnt family member 2B
YBX1	Y-box binding protein 1
YY1	Yin and yang 1
ZEB1	Zinc finger E-box binding homeobox 1
ZEB2	Zinc finger E-box-binding homeobox 2

## References

[1] Tirpe A.A., Gulei D., Ciortea S.M., Crivii C., Berindan-Neagoe I. (2019). Hypoxia: Overview on hypoxia-mediated mechanisms with a focus on the role of hif genes. Int. J. Mol. Sci..

[2] Sorensen B.S., Horsman M.R. (2020). Tumor hypoxia: Impact on radiation therapy and molecular pathways. Front. Oncol..

[3] Xue X., Jungles K., Onder G., Samhoun J., Gyorffy B., Hardiman K.M. (2016). Hif-3alpha1 promotes colorectal tumor cell growth by activation of jak-stat3 signaling. Oncotarget.

[4] Mizukami Y., Kohgo Y., Chung D.C. (2007). Hypoxia inducible factor-1 independent pathways in tumor angiogenesis. Clin. Cancer Res..

[5] Arsham A.M., Howell J.J., Simon M.C. (2003). A novel hypoxia-inducible factor-independent hypoxic response regulating mammalian target of rapamycin and its targets. J. Biol. Chem..

[6] Iommarini L., Porcelli A.M., Gasparre G., Kurelac I. (2017). Non-canonical mechanisms regulating hypoxia-inducible factor 1 alpha in cancer. Front. Oncol..

[7] Pahlman S., Mohlin S. (2018). Hypoxia and hypoxia-inducible factors in neuroblastoma. Cell Tissue Res..

[8] Flamant L., Notte A., Ninane N., Raes M., Michiels C. (2010). Anti-apoptotic role of hif-1 and ap-1 in paclitaxel exposed breast cancer cells under hypoxia. Mol. Cancer.

[9] Erler J.T., Cawthorne C.J., Williams K.J., Koritzinsky M., Wouters B.G., Wilson C., Miller C., Demonacos C., Stratford I.J., Dive C. (2004). Hypoxia-mediated down-regulation of bid and bax in tumors occurs via hypoxia-inducible factor 1-dependent and -independent mechanisms and contributes to drug resistance. Mol. Cell. Biol..

[10] Bertout J.A., Majmundar A.J., Gordan J.D., Lam J.C., Ditsworth D., Keith B., Brown E.J., Nathanson K.L., Simon M.C. (2009). Hif2alpha inhibition promotes p53 pathway activity, tumor cell death, and radiation responses. Proc. Natl. Acad. Sci. USA.

[11] Nardinocchi L., Puca R., D’Orazi G. (2011). Hif-1alpha antagonizes p53-mediated apoptosis by triggering hipk2 degradation. Aging.

[12] Zhang L., Huang G., Li X., Zhang Y., Jiang Y., Shen J., Liu J., Wang Q., Zhu J., Feng X. (2013). Hypoxia induces epithelial-mesenchymal transition via activation of snai1 by hypoxia-inducible factor -1alpha in hepatocellular carcinoma. BMC Cancer.

[13] Yang J., Zhang X., Zhang Y., Zhu D., Zhang L., Li Y., Zhu Y., Li D., Zhou J. (2016). Hif-2alpha promotes epithelial-mesenchymal transition through regulating twist2 binding to the promoter of e-cadherin in pancreatic cancer. J. Exp. Clin. Cancer Res..

[14] Zhang Q., Lou Y., Zhang J., Fu Q., Wei T., Sun X., Chen Q., Yang J., Bai X., Liang T. (2017). Hypoxia-inducible factor-2alpha promotes tumor progression and has crosstalk with wnt/beta-catenin signaling in pancreatic cancer. Mol. Cancer.

[15] Guo J., Wang B., Fu Z., Wei J., Lu W. (2016). Hypoxic microenvironment induces emt and upgrades stem-like properties of gastric cancer cells. Technol. Cancer Res. Treat..

[16] Liu Z., Tu K., Wang Y., Yao B., Li Q., Wang L., Dou C., Liu Q., Zheng X. (2017). Hypoxia accelerates aggressiveness of hepatocellular carcinoma cells involving oxidative stress, epithelial-mesenchymal transition and non-canonical hedgehog signaling. Cell. Physiol. Biochem..

[17] Son S.W., Song M.G., Yun B.D., Park J.K. (2021). Noncoding rnas associated with therapeutic resistance in pancreatic cancer. Biomedicines.

[18] Seo H.A., Moeng S., Sim S., Kuh H.J., Choi S.Y., Park J.K. (2019). Microrna-based combinatorial cancer therapy: Effects of micrornas on the efficacy of anti-cancer therapies. Cells.

[19] De Francesco E.M., Lappano R., Santolla M.F., Marsico S., Caruso A., Maggiolini M. (2013). Hif-1alpha/gper signaling mediates the expression of vegf induced by hypoxia in breast cancer associated fibroblasts (cafs). Breast Cancer Res..

[20] Morfoisse F., Kuchnio A., Frainay C., Gomez-Brouchet A., Delisle M.B., Marzi S., Helfer A.C., Hantelys F., Pujol F., Guillermet-Guibert J. (2014). Hypoxia induces vegf-c expression in metastatic tumor cells via a hif-1alpha-independent translation-mediated mechanism. Cell Rep..

[21] Tang N., Wang L., Esko J., Giordano F.J., Huang Y., Gerber H.P., Ferrara N., Johnson R.S. (2004). Loss of hif-1alpha in endothelial cells disrupts a hypoxia-driven vegf autocrine loop necessary for tumorigenesis. Cancer Cell.

[22] Garrido P., Osorio F.G., Moran J., Cabello E., Alonso A., Freije J.M., Gonzalez C. (2015). Loss of glut4 induces metabolic reprogramming and impairs viability of breast cancer cells. J. Cell. Physiol..

[23] Lu C.W., Lin S.C., Chen K.F., Lai Y.Y., Tsai S.J. (2008). Induction of pyruvate dehydrogenase kinase-3 by hypoxia-inducible factor-1 promotes metabolic switch and drug resistance. J. Biol. Chem..

[24] Chae Y.C., Vaira V., Caino M.C., Tang H.Y., Seo J.H., Kossenkov A.V., Ottobrini L., Martelli C., Lucignani G., Bertolini I. (2016). Mitochondrial akt regulation of hypoxic tumor reprogramming. Cancer Cell.

[25] Peng F., Wang J.H., Fan W.J., Meng Y.T., Li M.M., Li T.T., Cui B., Wang H.F., Zhao Y., An F. (2018). Glycolysis gatekeeper pdk1 reprograms breast cancer stem cells under hypoxia. Oncogene.

[26] Milane L., Duan Z., Amiji M. (2011). Role of hypoxia and glycolysis in the development of multi-drug resistance in human tumor cells and the establishment of an orthotopic multi-drug resistant tumor model in nude mice using hypoxic pre-conditioning. Cancer Cell Int..

[27] Marchiq I., Pouyssegur J. (2016). Hypoxia, cancer metabolism and the therapeutic benefit of targeting lactate/h(+) symporters. J. Mol. Med..

[28] Elia A.R., Cappello P., Puppo M., Fraone T., Vanni C., Eva A., Musso T., Novelli F., Varesio L., Giovarelli M. (2008). Human dendritic cells differentiated in hypoxia down-modulate antigen uptake and change their chemokine expression profile. J. Leukoc. Biol..

[29] Vito A., El-Sayes N., Mossman K. (2020). Hypoxia-driven immune escape in the tumor microenvironment. Cells.

[30] Betts G., Jones E., Junaid S., El-Shanawany T., Scurr M., Mizen P., Kumar M., Jones S., Rees B., Williams G. (2012). Suppression of tumour-specific cd4(+) t cells by regulatory t cells is associated with progression of human colorectal cancer. Gut.

[31] Togashi Y., Shitara K., Nishikawa H. (2019). Regulatory t cells in cancer immunosuppression—Implications for anticancer therapy. Nat. Rev. Clin. Oncol..

[32] Smyth M.J., Teng M.W., Swann J., Kyparissoudis K., Godfrey D.I., Hayakawa Y. (2006). Cd4+cd25+ t regulatory cells suppress nk cell-mediated immunotherapy of cancer. J. Immunol..

[33] Ren L., Yu Y., Wang L., Zhu Z., Lu R., Yao Z. (2016). Hypoxia-induced ccl28 promotes recruitment of regulatory t cells and tumor growth in liver cancer. Oncotarget.

[34] Deng B., Zhu J.M., Wang Y., Liu T.T., Ding Y.B., Xiao W.M., Lu G.T., Bo P., Shen X.Z. (2013). Intratumor hypoxia promotes immune tolerance by inducing regulatory t cells via tgf-beta1 in gastric cancer. PLoS ONE.

[35] Song Y., Wang R., Li L.W., Liu X., Wang Y.F., Wang Q.X., Zhang Q. (2019). Long non-coding rna hotair mediates the switching of histone h3 lysine 27 acetylation to methylation to promote epithelial-to-mesenchymal transition in gastric cancer. Int. J. Oncol..

[36] Hung T., Wang Y., Lin M.F., Koegel A.K., Kotake Y., Grant G.D., Horlings H.M., Shah N., Umbricht C., Wang P. (2011). Extensive and coordinated transcription of noncoding rnas within cell-cycle promoters. Nat. Genet..

[37] Yu S., Li N., Huang Z., Chen R., Yi P., Kang R., Tang D., Hu X., Fan X. (2018). A novel lncrna, tcons_00006195, represses hepatocellular carcinoma progression by inhibiting enzymatic activity of eno1. Cell Death Dis..

[38] Zhu J., Liu S., Ye F., Shen Y., Tie Y., Zhu J., Wei L., Jin Y., Fu H., Wu Y. (2015). Long noncoding rna meg3 interacts with p53 protein and regulates partial p53 target genes in hepatoma cells. PLoS ONE.

[39] Wang W., Hu W., Wang Y., An Y., Song L., Shang P., Yue Z. (2020). Long non-coding rna uca1 promotes malignant phenotypes of renal cancer cells by modulating the mir-182-5p/dll4 axis as a cerna. Mol. Cancer.

[40] Denzler R., McGeary S.E., Title A.C., Agarwal V., Bartel D.P., Stoffel M. (2016). Impact of microrna levels, target-site complementarity, and cooperativity on competing endogenous rna-regulated gene expression. Mol. Cell.

[41] Thomson D.W., Dinger M.E. (2016). Endogenous microrna sponges: Evidence and controversy. Nat. Rev. Genet..

[42] Kang X., Kong F., Wu S., Liu Q., Yang C., Wu X., Zhang W. (2019). Microrna-612 suppresses the malignant development of non-small-cell lung cancer by directly targeting bromodomain-containing protein 4. OncoTargets Ther..

[43] Sheng L., He P., Yang X., Zhou M., Feng Q. (2015). Mir-612 negatively regulates colorectal cancer growth and metastasis by targeting akt2. Cell Death Dis..

[44] Jin Y., Zhou X., Yao X., Zhang Z., Cui M., Lin Y. (2020). Microrna-612 inhibits cervical cancer progression by targeting nob1. J. Cell. Mol. Med..

[45] Yu A., Zhao L., Kang Q., Li J., Chen K., Fu H. (2020). Transcription factor hif1alpha promotes proliferation, migration, and invasion of cholangiocarcinoma via long noncoding rna h19/microrna-612/bcl-2 axis. Transl. Res..

[46] Chen L., Bao L., Niu Y., Wang J.E., Kumar A., Xing C., Wang Y., Luo W. (2021). Lncihat is induced by hypoxia-inducible factor 1 and promotes breast cancer progression. Mol. Cancer Res..

[47] Liu Z., Wang Y., Wang L., Yao B., Sun L., Liu R., Chen T., Niu Y., Tu K., Liu Q. (2019). Long non-coding rna agap2-as1, functioning as a competitive endogenous rna, upregulates anxa11 expression by sponging mir-16-5p and promotes proliferation and metastasis in hepatocellular carcinoma. J. Exp. Clin. Cancer Res..

[48] Liang Y., Song X., Li Y., Chen B., Zhao W., Wang L., Zhang H., Liu Y., Han D., Zhang N. (2020). Lncrna bcrt1 promotes breast cancer progression by targeting mir-1303/ptbp3 axis. Mol. Cancer.

[49] Zhang Z., Fang E., Rong Y., Han H., Gong Q., Xiao Y., Li H., Mei P., Li H., Zhu Z. (2021). Hypoxia-induced lncrna casc9 enhances glycolysis and the epithelial-mesenchymal transition of pancreatic cancer by a positive feedback loop with akt/hif-1alpha signaling. Am. J. Cancer Res..

[50] Yang X., Yao B., Niu Y., Chen T., Mo H., Wang L., Guo C., Yao D. (2019). Hypoxia-induced lncrna eif3j-as1 accelerates hepatocellular carcinoma progression via targeting mir-122-5p/ctnnd2 axis. Biochem. Biophys. Res. Commun..

[51] Ou Z.L., Zhang M., Ji L.D., Luo Z., Han T., Lu Y.B., Li Y.X. (2019). Long noncoding rna fezf1-as1 predicts poor prognosis and modulates pancreatic cancer cell proliferation and invasion through mir-142/hif-1alpha and mir-133a/egfr upon hypoxia/normoxia. J. Cell. Physiol..

[52] Wu W., Hu Q., Nie E., Yu T., Wu Y., Zhi T., Jiang K., Shen F., Wang Y., Zhang J. (2017). Hypoxia induces h19 expression through direct and indirect hif-1alpha activity, promoting oncogenic effects in glioblastoma. Sci. Rep..

[53] Xu Z., Lv H., Wang Y., Hu C., Chen S., Du Y., Shi C., Cheng X. (2020). Hand2-as1 inhibits gastric adenocarcinoma cells proliferation and aerobic glycolysis via mirnas sponge. Cancer Manag. Res..

[54] Mineo M., Ricklefs F., Rooj A.K., Lyons S.M., Ivanov P., Ansari K.I., Nakano I., Chiocca E.A., Godlewski J., Bronisz A. (2016). The long non-coding rna hif1a-as2 facilitates the maintenance of mesenchymal glioblastoma stem-like cells in hypoxic niches. Cell Rep..

[55] Chen X., Liu M., Meng F., Sun B., Jin X., Jia C. (2019). The long noncoding rna hif1a-as2 facilitates cisplatin resistance in bladder cancer. J. Cell. Biochem..

[56] Shih J.W., Chiang W.F., Wu A.T.H., Wu M.H., Wang L.Y., Yu Y.L., Hung Y.W., Wang W.C., Chu C.Y., Hung C.L. (2017). Long noncoding rna lnchifcar/mir31hg is a hif-1alpha co-activator driving oral cancer progression. Nat. Commun..

[57] Wang X., Wang Y., Li L., Xue X., Xie H., Shi H., Hu Y. (2020). A lncrna coordinates with ezh2 to inhibit hif-1alpha transcription and suppress cancer cell adaption to hypoxia. Oncogene.

[58] Wang X., Li L., Zhao K., Lin Q., Li H., Xue X., Ge W., He H., Liu D., Xie H. (2020). A novel lncrna hitt forms a regulatory loop with hif-1alpha to modulate angiogenesis and tumor growth. Cell Death Differ..

[59] Hu M., Fu Q., Jing C., Zhang X., Qin T., Pan Y. (2020). Lncrna hotair knockdown inhibits glycolysis by regulating mir-130a-3p/hif1a in hepatocellular carcinoma under hypoxia. Biomed. Pharmacother..

[60] Zhang S., Wang W., Liu G., Xie S., Li Q., Li Y., Lin Z. (2017). Long non-coding rna hottip promotes hypoxia-induced epithelial-mesenchymal transition of malignant glioma by regulating the mir-101/zeb1 axis. Biomed. Pharmacother..

[61] Shi J., Wang H., Feng W., Huang S., An J., Qiu Y., Wu K. (2019). Long non-coding rna hottip promotes hypoxia-induced glycolysis through targeting mir-615-3p/hmgb3 axis in non-small cell lung cancer cells. Eur. J. Pharmacol..

[62] Zhu P., He F., Hou Y., Tu G., Li Q., Jin T., Zeng H., Qin Y., Wan X., Qiao Y. (2021). A novel hypoxic long noncoding rna kb-1980e6.3 maintains breast cancer stem cell stemness via interacting with igf2bp1 to facilitate c-myc mrna stability. Oncogene.

[63] Yu L., Gui S., Liu Y., Qiu X., Qiu B., Zhang X., Pan J., Fan J., Qi S., Zhang G. (2020). Long intergenic non-protein coding rna 00475 silencing acts as a tumor suppressor in glioma under hypoxic condition by impairing microrna-449b-5p-dependent agap2 up-regulation. Ther. Adv. Med. Oncol..

[64] Sun S., Xia C., Xu Y. (2020). Hif-1alpha induced lncrna linc00511 accelerates the colorectal cancer proliferation through positive feedback loop. Biomed. Pharmacother..

[65] Yuan S., Xiang Y., Wang G., Zhou M., Meng G., Liu Q., Hu Z., Li C., Xie W., Wu N. (2019). Hypoxia-sensitive linc01436 is regulated by e2f6 and acts as an oncogene by targeting mir-30a-3p in non-small cell lung cancer. Mol. Oncol..

[66] Zhao Z.B., Chen F., Bai X.F. (2019). Long noncoding rna malat1 regulates hepatocellular carcinoma growth under hypoxia via sponging microrna-200a. Yonsei Med. J..

[67] Wang L., Sun L., Liu R., Mo H., Niu Y., Chen T., Wang Y., Han S., Tu K., Liu Q. (2021). Long non-coding rna mapkapk5-as1/plagl2/hif-1alpha signaling loop promotes hepatocellular carcinoma progression. J. Exp. Clin. Cancer Res..

[68] Kong X., Zhao Y., Li X., Tao Z., Hou M., Ma H. (2019). Overexpression of hif-2alpha-dependent neat1 promotes the progression of non-small cell lung cancer through mir-101-3p/sox9/wnt/beta-catenin signal pathway. Cell. Physiol. Biochem..

[69] Tan X., Wang P., Lou J., Zhao J. (2020). Knockdown of lncrna neat1 suppresses hypoxia-induced migration, invasion and glycolysis in anaplastic thyroid carcinoma cells through regulation of mir-206 and mir-599. Cancer Cell Int..

[70] Li H., Wang X., Wen C., Huo Z., Wang W., Zhan Q., Cheng D., Chen H., Deng X., Peng C. (2017). Long noncoding rna norad, a novel competing endogenous rna, enhances the hypoxia-induced epithelial-mesenchymal transition to promote metastasis in pancreatic cancer. Mol. Cancer.

[71] He H., Chen T., Mo H., Chen S., Liu Q., Guo C. (2020). Hypoxia-inducible long noncoding rna npsr1-as1 promotes the proliferation and glycolysis of hepatocellular carcinoma cells by regulating the mapk/erk pathway. Biochem. Biophys. Res. Commun..

[72] Li X., Deng S.J., Zhu S., Jin Y., Cui S.P., Chen J.Y., Xiang C., Li Q.Y., He C., Zhao S.F. (2016). Hypoxia-induced lncrna-nutf2p3-001 contributes to tumorigenesis of pancreatic cancer by derepressing the mir-3923/kras pathway. Oncotarget.

[73] Niu Y., Bao L., Chen Y., Wang C., Luo M., Zhang B., Zhou M., Wang J.E., Fang Y.V., Kumar A. (2020). Hif2-induced long noncoding rna rab11b-as1 promotes hypoxia-mediated angiogenesis and breast cancer metastasis. Cancer Res..

[74] Peng P.H., Chieh-Yu Lai J., Hsu K.W., Wu K.J. (2020). Hypoxia-induced lncrna rp11-390f4.3 promotes epithelial-mesenchymal transition (emt) and metastasis through upregulating emt regulators. Cancer Lett..

[75] Yang Z., Shi X., Li C., Wang X., Hou K., Li Z., Zhang X., Fan Y., Qu X., Che X. (2018). Long non-coding rna uca1 upregulation promotes the migration of hypoxia-resistant gastric cancer cells through the mir-7-5p/egfr axis. Exp. Cell Res..

[76] Zhao C.H., Bai X.F., Hu X.H. (2020). Knockdown of lncrna xist inhibits hypoxia-induced glycolysis, migration and invasion through regulating mir-381-3p/nek5 axis in nasopharyngeal carcinoma. Eur. Rev. Med. Pharmacol. Sci..

[77] Gan L., Yang Y., Li Q., Feng Y., Liu T., Guo W. (2018). Epigenetic regulation of cancer progression by ezh2: From biological insights to therapeutic potential. Biomark. Res..

[78] Ahn J.Y., Rong R., Liu X., Ye K. (2004). Pike/nuclear pi 3-kinase signaling mediates the antiapoptotic actions of ngf in the nucleus. EMBO J..

[79] Qi Q., Ye K. (2013). The roles of pike in tumorigenesis. Acta Pharmacol. Sin..

[80] Hou W.Z., Chen X.L., Qin L.S., Xu Z.J., Liao G.M., Chen D., Hu L.J., Mao Z.M., Huang J.-S., Yuan Q. (2020). Mir-449b-5p inhibits human glioblastoma cell proliferation by inactivating wnt2b/wnt/beta-catenin signaling pathway. Eur. Rev. Med. Pharmacol. Sci..

[81] Jiang J., Yang X., He X., Ma W., Wang J., Zhou Q., Li M., Yu S. (2019). Microrna-449b-5p suppresses the growth and invasion of breast cancer cells via inhibiting crept-mediated wnt/beta-catenin signaling. Chem. Biol. Interact..

[82] Yu J., Wang F., Zhang J., Li J., Chen X., Han G. (2020). Linc00667/mir-449b-5p/yy1 axis promotes cell proliferation and migration in colorectal cancer. Cancer Cell Int..

[83] Guo Q., Wang T., Yang Y., Gao L., Zhao Q., Zhang W., Xi T., Zheng L. (2020). Transcriptional factor yin yang 1 promotes the stemness of breast cancer cells by suppressing mir-873-5p transcriptional activity. Mol. Ther. Nucleic Acids.

[84] Yang L., Yang H., Chu Y., Song Y., Ding L., Zhu B., Zhai W., Wang X., Kuang Y., Ren F. (2021). Crept is required for murine stem cell maintenance during intestinal regeneration. Nat. Commun..

[85] Kim J.H., Park S.Y., Jun Y., Kim J.Y., Nam J.S. (2017). Roles of wnt target genes in the journey of cancer stem cells. Int. J. Mol. Sci..

[86] Shi G., Cheng Y., Zhang Y., Guo R., Li S., Hong X. (2021). Long non-coding rna linc00511/mir-150/mmp13 axis promotes breast cancer proliferation, migration and invasion. Biochim. Biophys. Acta Mol. Basis Dis..

[87] Jiang L., Xie X., Bi R., Ding F., Mei J. (2020). Knockdown of linc00511 inhibits tgf-beta-induced cell migration and invasion by suppressing epithelial-mesenchymal transition and down-regulating mmps expression. Biomed. Pharmacother..

[88] Zhao X., Liu Y., Li Z., Zheng S., Wang Z., Li W., Bi Z., Li L., Jiang Y., Luo Y. (2018). Linc00511 acts as a competing endogenous rna to regulate vegfa expression through sponging hsa-mir-29b-3p in pancreatic ductal adenocarcinoma. J. Cell. Mol. Med..

[89] Hu Y., Zhang Y., Ding M., Xu R. (2020). Lncrna linc00511 acts as an oncogene in colorectal cancer via sponging mir-29c-3p to upregulate nfia. OncoTargets Ther..

[90] Lu Y., Yu Y., Liu F., Han Y., Xue H., Sun X., Jiang Y., Tian Z. (2021). Linc00511-dependent inhibition of il-24 contributes to the oncogenic role of hnf4alpha in colorectal cancer. Am. J. Physiol. Gastrointest. Liver Physiol..

[91] He Y., Zhang L., Tan F., Wang L.F., Liu D.H., Wang R.J., Yin X.Z. (2020). Mir-153-5p promotes sensibility of colorectal cancer cells to oxaliplatin via targeting bcl-2-mediated autophagy pathway. Biosci. Biotechnol. Biochem..

[92] Kim J., Piao H.L., Kim B.J., Yao F., Han Z., Wang Y., Xiao Z., Siverly A.N., Lawhon S.E., Ton B.N. (2018). Long noncoding rna malat1 suppresses breast cancer metastasis. Nat. Genet..

[93] Li L., Chen H., Gao Y., Wang Y.W., Zhang G.Q., Pan S.H., Ji L., Kong R., Wang G., Jia Y.H. (2016). Long noncoding rna malat1 promotes aggressive pancreatic cancer proliferation and metastasis via the stimulation of autophagy. Mol. Cancer Ther..

[94] Peng N., He J., Li J., Huang H., Huang W., Liao Y., Zhu S. (2020). Long noncoding rna malat1 inhibits the apoptosis and autophagy of hepatocellular carcinoma cell by targeting the microrna-146a/pi3k/akt/mtor axis. Cancer Cell Int..

[95] Liang X.L., Wang Y.L., Wang P.R. (2021). Mir-200a with cdc7 as a direct target declines cell viability and promotes cell apoptosis in wilm’s tumor via wnt/beta-catenin signaling pathway. Mol. Cell. Biochem..

[96] Ba M.C., Ba Z., Long H., Cui S.Z., Gong Y.F., Yan Z.F., Lin K.P., Wu Y.B., Tu Y.N. (2020). Lncrna ac093818.1 accelerates gastric cancer metastasis by epigenetically promoting pdk1 expression. Cell Death Dis..

[97] Gaudreault M., Vigneault F., Gingras M.E., Leclerc S., Carrier P., Germain L., Guerin S.L. (2008). Transcriptional regulation of the human alpha6 integrin gene by the transcription factor nfi during corneal wound healing. Investig. Ophthalmol. Vis. Sci..

[98] Wang M., Dong Q., Zhang D., Wang Y. (2011). Expression of delta-catenin is associated with progression of human astrocytoma. BMC Cancer.

[99] Nopparat J., Zhang J., Lu J.P., Chen Y.H., Zheng D., Neufer P.D., Fan J.M., Hong H., Boykin C., Lu Q. (2015). Delta-catenin, a wnt/beta-catenin modulator, reveals inducible mutagenesis promoting cancer cell survival adaptation and metabolic reprogramming. Oncogene.

[100] Huang F., Chen J., Wang Z., Lan R., Fu L., Zhang L. (2018). Delta-catenin promotes tumorigenesis and metastasis of lung adenocarcinoma. Oncol. Rep..

[101] Hou P., Li L., Chen F., Chen Y., Liu H., Li J., Bai J., Zheng J. (2018). Ptbp3-mediated regulation of zeb1 mrna stability promotes epithelial-mesenchymal transition in breast cancer. Cancer Res..

[102] Hou P., Chen F., Yong H., Lin T., Li J., Pan Y., Jiang T., Li M., Chen Y., Song J. (2019). Ptbp3 contributes to colorectal cancer growth and metastasis via translational activation of hif-1alpha. J. Exp. Clin. Cancer Res..

[103] Ma J., Weng L., Jia Y., Liu B., Wu S., Xue L., Yin X., Mao A., Wang Z., Shang M. (2020). Ptbp3 promotes malignancy and hypoxia-induced chemoresistance in pancreatic cancer cells by atg12 up-regulation. J. Cell. Mol. Med..

[104] Hui Y., Yang Y., Li D., Wang J., Di M., Zhang S., Wang S. (2020). Lncrna fezf1-as1 modulates cancer stem cell properties of human gastric cancer through mir-363-3p/hmga2. Cell Transplant..

[105] Wang Y.D., Sun X.J., Yin J.J., Yin M., Wang W., Nie Z.Q., Xu J. (2018). Long non-coding rna fezf1-as1 promotes cell invasion and epithelial-mesenchymal transition through jak2/stat3 signaling pathway in human hepatocellular carcinoma. Biomed. Pharmacother..

[106] Huang S., Li C., Huang J., Luo P., Mo D., Wang H. (2020). Lncrna fezf1-as1 promotes non-small lung cancer cell migration and invasion through the up-regulation of notch1 by serving as a sponge of mir-34a. BMC Pulm. Med..

[107] Bian Z., Zhang J., Li M., Feng Y., Wang X., Zhang J., Yao S., Jin G., Du J., Han W. (2018). Lncrna-fezf1-as1 promotes tumor proliferation and metastasis in colorectal cancer by regulating pkm2 signaling. Clin. Cancer Res..

[108] Ye H., Zhou Q., Zheng S., Li G., Lin Q., Ye L., Wang Y., Wei L., Zhao X., Li W. (2018). Fezf1-as1/mir-107/znf312b axis facilitates progression and warburg effect in pancreatic ductal adenocarcinoma. Cell Death Dis..

[109] Li L., Shao M.Y., Zou S.C., Xiao Z.F., Chen Z.C. (2019). Mir-101-3p inhibits emt to attenuate proliferation and metastasis in glioblastoma by targeting trim44. J. Neurooncol..

[110] Ji H., Hui B., Wang J., Zhu Y., Tang L., Peng P., Wang T., Wang L., Xu S., Li J. (2019). Long noncoding rna mapkapk5-as1 promotes colorectal cancer proliferation by partly silencing p21 expression. Cancer Sci..

[111] Yang T., Chen W.C., Shi P.C., Liu M.R., Jiang T., Song H., Wang J.Q., Fan R.Z., Pei D.S., Song J. (2020). Long noncoding rna mapkapk5-as1 promotes colorectal cancer progression by cis-regulating the nearby gene mk5 and acting as a let-7f-1-3p sponge. J. Exp. Clin. Cancer Res..

[112] Zhou Y., Liu S., Luo Y., Zhang M., Jiang X., Xiong Y. (2020). Incrna mapkapk5-as1 promotes proliferation and migration of thyroid cancer cell lines by targeting mir-519e-5p/ywhah. Eur. J. Histochem..

[113] Tan B.S., Yang M.C., Singh S., Chou Y.C., Chen H.Y., Wang M.Y., Wang Y.C., Chen R.H. (2019). Lncrna norad is repressed by the yap pathway and suppresses lung and breast cancer metastasis by sequestering s100p. Oncogene.

[114] Gong P., Qiao F., Wu H., Cui H., Li Y., Zheng Y., Zhou M., Fan H. (2018). Lncrna uca1 promotes tumor metastasis by inducing mir-203/zeb2 axis in gastric cancer. Cell Death Dis..

[115] Luan Y., Li X., Luan Y., Zhao R., Li Y., Liu L., Hao Y., Oleg Vladimir B., Jia L. (2020). Circulating lncrna uca1 promotes malignancy of colorectal cancer via the mir-143/myo6 axis. Mol. Ther. Nucleic Acids.

[116] Chen Z., Liu Z., Yang Y., Zhu Z., Liang R., Huang B., Wu D., Yang L., Lu H., Jin D. (2018). Long non-coding rna rab11b-as1 prevents osteosarcoma development and progression via its natural antisense transcript rab11b. Oncotarget.

[117] Puca F., Tosti N., Federico A., Kuzay Y., Pepe A., Morlando S., Savarese T., D’Alessio F., Colamaio M., Sarnataro D. (2019). Hmga1 negatively regulates numb expression at transcriptional and post transcriptional level in glioblastoma stem cells. Cell Cycle.

[118] Elcheva I.A., Wood T., Chiarolanzio K., Chim B., Wong M., Singh V., Gowda C.P., Lu Q., Hafner M., Dovat S. (2020). Rna-binding protein igf2bp1 maintains leukemia stem cell properties by regulating hoxb4, myb, and aldh1a1. Leukemia.

[119] Wang Y., Zhang J., Su Y., Wang C., Zhang G., Liu X., Chen Q., Lv M., Chang Y., Peng J. (2020). Mirna-98-5p targeting igf2bp1 induces mesenchymal stem cell apoptosis by modulating pi3k/akt and p53 in immune thrombocytopenia. Mol. Ther. Nucleic Acids.

[120] Weidensdorfer D., Stohr N., Baude A., Lederer M., Kohn M., Schierhorn A., Buchmeier S., Wahle E., Huttelmaier S. (2009). Control of c-myc mrna stability by igf2bp1-associated cytoplasmic rnps. RNA.

[121] Liu H., Li C., Yang J., Sun Y., Zhang S., Yang J., Yang L., Wang Y., Jiao B. (2018). Long noncoding rna casc9/mir-519d/stat3 positive feedback loop facilitate the glioma tumourigenesis. J. Cell. Mol. Med..

[122] Luo K., Geng J., Zhang Q., Xu Y., Zhou X., Huang Z., Shi K.Q., Pan C., Wu J. (2019). Lncrna casc9 interacts with cpsf3 to regulate tgf-beta signaling in colorectal cancer. J. Exp. Clin. Cancer Res..

[123] Chen Z., Chen Q., Cheng Z., Gu J., Feng W., Lei T., Huang J., Pu J., Chen X., Wang Z. (2020). Long non-coding rna casc9 promotes gefitinib resistance in nsclc by epigenetic repression of dusp1. Cell Death Dis..

[124] Chen Y., Li Y., Gao H. (2020). Long noncoding rna casc9 promotes the proliferation and metastasis of papillary thyroid cancer via sponging mir-488-3p. Cancer Med..

[125] Gokulnath P., de Cristofaro T., Manipur I., Di Palma T., Soriano A.A., Guarracino M.R., Zannini M. (2020). Long non-coding rna hand2-as1 acts as a tumor suppressor in high-grade serous ovarian carcinoma. Int. J. Mol. Sci..

[126] Dong G., Wang X., Jia Y., Jia Y., Zhao W., Zhang J., Tong Z. (2020). Hand2-as1 works as a cerna of mir-3118 to suppress proliferation and migration in breast cancer by upregulating phlpp2. BioMed Res. Int..

[127] Chen S., Wang J. (2019). Hand2-as1 inhibits invasion and metastasis of cervical cancer cells via microrna-330-5p-mediated ldoc1. Cancer Cell Int..

[128] Yang W., Zheng Y., Xia Y., Ji H., Chen X., Guo F., Lyssiotis C.A., Aldape K., Cantley L.C., Lu Z. (2012). Erk1/2-dependent phosphorylation and nuclear translocation of pkm2 promotes the warburg effect. Nat. Cell Biol..

[129] Chen C., Peng S., Li P., Ma L., Gan X. (2020). High expression of nek2 promotes lung cancer progression and drug resistance and is regulated by mutant egfr. Mol. Cell. Biochem..

[130] Gu Z., Xia J., Xu H., Frech I., Tricot G., Zhan F. (2017). Nek2 promotes aerobic glycolysis in multiple myeloma through regulating splicing of pyruvate kinase. J. Hematol. Oncol..

[131] Liu J., Wang L., Li X. (2018). Hmgb3 promotes the proliferation and metastasis of glioblastoma and is negatively regulated by mir-200b-3p and mir-200c-3p. Cell Biochem. Funct..

[132] Zhou Y., Lin F., Wan T., Chen A., Wang H., Jiang B., Zhao W., Liao S., Wang S., Li G. (2021). Zeb1 enhances warburg effect to facilitate tumorigenesis and metastasis of hcc by transcriptionally activating pfkm. Theranostics.

[133] Mo Y., Wang Y., Zhang L., Yang L., Zhou M., Li X., Li Y., Li G., Zeng Z., Xiong W. (2019). The role of wnt signaling pathway in tumor metabolic reprogramming. J. Cancer.

[134] Ferezin C.C., Basei F.L., Melo-Hanchuk T.D., de Oliveira A.L., Peres de Oliveira A., Mori M.P., de Souza-Pinto N.C., Kobarg J. (2021). Nek5 interacts with lonp1 and its kinase activity is essential for the regulation of mitochondrial functions and mtdna maintenance. FEBS Open Bio.

[135] Luo B., Wang M., Hou N., Hu X., Jia G., Qin X., Zuo X., Liu Y., Luo K., Song W. (2016). Atp-dependent lon protease contributes to helicobacter pylori-induced gastric carcinogenesis. Neoplasia.

[136] Wang G., Xu G., Wang W. (2020). Long noncoding rna cdkn2b-as1 facilitates lung cancer development through regulating mir-378b/nr2c2. OncoTargets Ther..

[137] Shen X., Li Y., He F., Kong J. (2020). Lncrna cdkn2b-as1 promotes cell viability, migration, and invasion of hepatocellular carcinoma via sponging mir-424-5p. Cancer Manag. Res..

[138] Wang Y., Huang Y., Liu H., Su D., Luo F., Zhou F. (2019). Long noncoding rna cdkn2b-as1 interacts with mir-411-3p to regulate ovarian cancer in vitro and in vivo through hif-1a/vegf/p38 pathway. Biochem. Biophys. Res. Commun..

[139] Khandrika L., Lieberman R., Koul S., Kumar B., Maroni P., Chandhoke R., Meacham R.B., Koul H.K. (2009). Hypoxia-associated p38 mitogen-activated protein kinase-mediated androgen receptor activation and increased hif-1alpha levels contribute to emergence of an aggressive phenotype in prostate cancer. Oncogene.

[140] Cheung S., Jain P., So J., Shahidi S., Chung S., Koritzinsky M. (2021). P38 mapk inhibition mitigates hypoxia-induced ar signaling in castration-resistant prostate cancer. Cancers.

[141] Liu A.M., Zhu Y., Huang Z.W., Lei L., Fu S.Z., Chen Y. (2019). Long noncoding rna fam201a involves in radioresistance of non-small-cell lung cancer by enhancing egfr expression via mir-370. Eur. Rev. Med. Pharmacol. Sci..

[142] Zhu H., Jin Y.M., Lyu X.M., Fan L.M., Wu F. (2019). Long noncoding rna h19 regulates hif-1alpha/axl signaling through inhibiting mir-20b-5p in endometrial cancer. Cell Cycle.

[143] Liu Z.Z., Tian Y.F., Wu H., Ouyang S.Y., Kuang W.L. (2020). Lncrna h19 promotes glioma angiogenesis through mir-138/hif-1alpha/vegf axis. Neoplasma.

[144] Hong Q., Li O., Zheng W., Xiao W.Z., Zhang L., Wu D., Cai G.Y., He J.C., Chen X.M. (2017). Lncrna hotair regulates hif-1alpha/axl signaling through inhibition of mir-217 in renal cell carcinoma. Cell Death Dis..

[145] Wang S., You H., Yu S. (2020). Long non-coding rna hoxa-as2 promotes the expression levels of hypoxia-inducible factor-1alpha and programmed death-ligand 1, and regulates nasopharyngeal carcinoma progression via mir-519. Oncol. Lett..

[146] Cai Q., Wang Z., Wang S., Weng M., Zhou D., Li C., Wang J., Chen E., Quan Z. (2017). Long non-coding rna linc00152 promotes gallbladder cancer metastasis and epithelial-mesenchymal transition by regulating hif-1alpha via mir-138. Open Biol..

[147] Sun C.C., Zhu W., Li S.J., Hu W., Zhang J., Zhuo Y., Zhang H., Wang J., Zhang Y., Huang S.X. (2020). Foxc1-mediated linc00301 facilitates tumor progression and triggers an immune-suppressing microenvironment in non-small cell lung cancer by regulating the hif1alpha pathway. Genome Med..

[148] Liu Y., He D., Xiao M., Zhu Y., Zhou J., Cao K. (2021). Long noncoding rna linc00518 induces radioresistance by regulating glycolysis through an mir-33a-3p/hif-1alpha negative feedback loop in melanoma. Cell Death Dis..

[149] Tan H., Zhao L. (2019). Lncrna nuclear-enriched abundant transcript 1 promotes cell proliferation and invasion by targeting mir-186-5p/hif-1alpha in osteosarcoma. J. Cell. Biochem..

[150] Du F., Guo T., Cao C. (2020). Silencing of long noncoding rna snhg6 inhibits esophageal squamous cell carcinoma progression via mir-186-5p/hif1alpha axis. Dig. Dis. Sci..

[151] Fan X., Zhao Z., Song J., Zhang D., Wu F., Tu J., Xu M., Ji J. (2021). Lncrna-snhg6 promotes the progression of hepatocellular carcinoma by targeting mir-6509-5p and hif1a. Cancer Cell Int..

[152] Zhao P., Deng Y., Wu Y., Guo Q., Zhou L., Yang X., Wang C. (2021). Long noncoding rna snhg6 promotes carcinogenesis by enhancing ybx1-mediated translation of hif1alpha in clear cell renal cell carcinoma. FASEB J..

[153] Xu L., Huan L., Guo T., Wu Y., Liu Y., Wang Q., Huang S., Xu Y., Liang L., He X. (2020). Lncrna snhg11 facilitates tumor metastasis by interacting with and stabilizing hif-1alpha. Oncogene.

[154] Peng X., Yan J., Cheng F. (2020). Lncrna tmpo-as1 up-regulates the expression of hif-1alpha and promotes the malignant phenotypes of retinoblastoma cells via sponging mir-199a-5p. Pathol. Res. Pract..

[155] Yu X., Hu L., Li S., Shen J., Wang D., Xu R., Yang H. (2019). Long non-coding rna taurine upregulated gene 1 promotes osteosarcoma cell metastasis by mediating hif-1alpha via mir-143-5p. Cell Death Dis..

[156] Li X., Wu Y., Liu A., Tang X. (2016). Long non-coding rna uca1 enhances tamoxifen resistance in breast cancer cells through a mir-18a-hif1alpha feedback regulatory loop. Tumour Biol..

[157] Yang L.G., Cao M.Z., Zhang J., Li X.Y., Sun Q.L. (2020). Lncrna xist modulates hif-1a/axl signaling pathway by inhibiting mir-93-5p in colorectal cancer. Mol. Genet. Genom. Med..

[158] Wu F., Gao H., Liu K., Gao B., Ren H., Li Z., Liu F. (2019). The lncrna zeb2-as1 is upregulated in gastric cancer and affects cell proliferation and invasion via mir-143-5p/hif-1alpha axis. OncoTargets Ther..

[159] Linger R.M., Cohen R.A., Cummings C.T., Sather S., Migdall-Wilson J., Middleton D.H., Lu X., Baron A.E., Franklin W.A., Merrick D.T. (2013). Mer or axl receptor tyrosine kinase inhibition promotes apoptosis, blocks growth and enhances chemosensitivity of human non-small cell lung cancer. Oncogene.

[160] Papadakis E.S., Cichon M.A., Vyas J.J., Patel N., Ghali L., Cerio R., Storey A., O’Toole E.A. (2011). Axl promotes cutaneous squamous cell carcinoma survival through negative regulation of pro-apoptotic bcl-2 family members. J. Investig. Dermatol..

[161] Rankin E.B., Fuh K.C., Castellini L., Viswanathan K., Finger E.C., Diep A.N., LaGory E.L., Kariolis M.S., Chan A., Lindgren D. (2014). Direct regulation of gas6/axl signaling by hif promotes renal metastasis through src and met. Proc. Natl. Acad. Sci. USA.

[162] Liang J., Liu Y., Zhang L., Tan J., Li E., Li F. (2019). Overexpression of microrna-519d-3p suppressed the growth of pancreatic cancer cells by inhibiting ribosomal protein s15a-mediated wnt/beta-catenin signaling. Chem. Biol. Interact..

[163] Yang H., Qi Y., Wang X.L., Gu J.J., Shi T.M. (2020). Down-regulation of lncrna blacat1 inhibits ovarian cancer progression by suppressing the wnt/beta-catenin signaling pathway via regulating mir-519d-3p. Mol. Cell. Biochem..

[164] Zhang G., Hu Y., Yuan W., Qiu H., Yu H., Du J. (2020). Mir-519d-3p overexpression inhibits p38 and pi3k/akt pathway via targeting vegfa to attenuate the malignant biological behavior of non-small cell lung cancer. OncoTargets Ther..

[165] Jiang L., Shi S., Shi Q., Zhang H., Xia Y., Zhong T. (2018). Microrna-519d-3p inhibits proliferation and promotes apoptosis by targeting hif-2alpha in cervical cancer under hypoxic conditions. Oncol. Res..

[166] Ding Y., Guo H., Zhu L., Xu L., Pei Q., Cao Y. (2020). Linc00152 knock-down suppresses esophageal cancer by egfr signaling pathway. Open Med..

[167] Zhang S., Liao W., Wu Q., Huang X., Pan Z., Chen W., Gu S., Huang Z., Wang Y., Tang X. (2020). Linc00152 upregulates zeb1 expression and enhances epithelial-mesenchymal transition and oxaliplatin resistance in esophageal cancer by interacting with ezh2. Cancer Cell Int..

[168] Li Q., Wang X., Zhou L., Jiang M., Zhong G., Xu S., Zhang M., Zhang Y., Liang X., Zhang L. (2021). A positive feedback loop of long noncoding rna linc00152 and klf5 facilitates breast cancer growth. Front. Oncol..

[169] Li X., Liu X., Xu Y., Liu J., Xie M., Ni W., Chen S. (2014). Klf5 promotes hypoxia-induced survival and inhibits apoptosis in non-small cell lung cancer cells via hif-1alpha. Int. J. Oncol..

[170] Zhou H., Li L., Wang Y., Wang D. (2020). Long non-coding rna snhg6 promotes tumorigenesis in melanoma cells via the microrna-101-3p/rap2b axis. Oncol. Lett..

[171] Shao Q., Xu J., Deng R., Wei W., Zhou B., Yue C., Zhu M., Zhu H. (2019). Snhg 6 promotes the progression of colon and rectal adenocarcinoma via mir-101-3p and wnt/beta-catenin signaling pathway. BMC Gastroenterol..

[172] Wang X., Lai Q., He J., Li Q., Ding J., Lan Z., Gu C., Yan Q., Fang Y., Zhao X. (2019). Lncrna snhg6 promotes proliferation, invasion and migration in colorectal cancer cells by activating tgf-beta/smad signaling pathway via targeting upf1 and inducing emt via regulation of zeb1. Int. J. Med. Sci..

[173] Liang R., Xiao G., Wang M., Li X., Li Y., Hui Z., Sun X., Qin S., Zhang B., Du N. (2018). Snhg6 functions as a competing endogenous rna to regulate e2f7 expression by sponging mir-26a-5p in lung adenocarcinoma. Biomed. Pharmacother..

[174] Kamura T., Sato S., Iwai K., Czyzyk-Krzeska M., Conaway R.C., Conaway J.W. (2000). Activation of hif1alpha ubiquitination by a reconstituted von hippel-lindau (vhl) tumor suppressor complex. Proc. Natl. Acad. Sci. USA.

[175] Yu F., White S.B., Zhao Q., Lee F.S. (2001). Hif-1alpha binding to vhl is regulated by stimulus-sensitive proline hydroxylation. Proc. Natl. Acad. Sci. USA.

[176] Huang W., Dong S., Cha Y., Yuan X. (2020). Snhg11 promotes cell proliferation in colorectal cancer by forming a positive regulatory loop with c-myc. Biochem. Biophys. Res. Commun..

[177] Doe M.R., Ascano J.M., Kaur M., Cole M.D. (2012). Myc posttranscriptionally induces hif1 protein and target gene expression in normal and cancer cells. Cancer Res..

[178] Sun N., Zhang G., Liu Y. (2018). Long non-coding rna xist sponges mir-34a to promotes colon cancer progression via wnt/beta-catenin signaling pathway. Gene.

[179] Xue F., Song X., Zhang S., Niu M., Cui Y., Wang Y., Zhao T. (2021). Long non-coding rna tmpo-as1 serves as a tumor promoter in pancreatic carcinoma by regulating mir-383-5p/sox11. Oncol. Lett..

[180] Hu Y., Zhang Y., Ding M., Xu R. (2020). Long noncoding rna tmpo-as1/mir-126-5p/brcc3 axis accelerates gastric cancer progression and angiogenesis via activating pi3k/akt/mtor pathway. J. Gastroenterol. Hepatol..

[181] Guo X., Wang Y. (2020). Lncrna tmpo-as1 promotes hepatocellular carcinoma cell proliferation, migration and invasion through sponging mir-329-3p to stimulate foxk1-mediated akt/mtor signaling pathway. Cancer Med..

[182] Diao P., Ge H., Song Y., Wu Y., Li J., Li Z., Yang J., Wang Y., Cheng J. (2019). Overexpression of zeb2-as1 promotes epithelial-to-mesenchymal transition and metastasis by stabilizing zeb2 mrna in head neck squamous cell carcinoma. J. Cell. Mol. Med..

[183] Zhang G., Li H., Sun R., Li P., Yang Z., Liu Y., Wang Z., Yang Y., Yin C. (2019). Long non-coding rna zeb2-as1 promotes the proliferation, metastasis and epithelial mesenchymal transition in triple-negative breast cancer by epigenetically activating zeb2. J. Cell. Mol. Med..

[184] Wang F., Zhu W., Yang R., Xie W., Wang D. (2019). Lncrna zeb2-as1 contributes to the tumorigenesis of gastric cancer via activating the wnt/beta-catenin pathway. Mol. Cell. Biochem..

[185] Jia P., Cai H., Liu X., Chen J., Ma J., Wang P., Liu Y., Zheng J., Xue Y. (2016). Long non-coding rna h19 regulates glioma angiogenesis and the biological behavior of glioma-associated endothelial cells by inhibiting microrna-29a. Cancer Lett..

[186] Zhou Q., Liu Z.Z., Wu H., Kuang W.L. (2020). Lncrna h19 promotes cell proliferation, migration, and angiogenesis of glioma by regulating wnt5a/beta-catenin pathway via targeting mir-342. Cell. Mol. Neurobiol..

[187] Lv M., Zhong Z., Huang M., Tian Q., Jiang R., Chen J. (2017). Lncrna h19 regulates epithelial-mesenchymal transition and metastasis of bladder cancer by mir-29b-3p as competing endogenous rna. Biochim. Biophys. Acta Mol. Cell Res..

[188] Huang S.M., Bock J.M., Harari P.M. (1999). Epidermal growth factor receptor blockade with c225 modulates proliferation, apoptosis, and radiosensitivity in squamous cell carcinomas of the head and neck. Cancer Res..

[189] Alexandru O., Purcaru S.O., Tataranu L.G., Lucan L., Castro J., Folcuti C., Artene S.A., Tuta C., Dricu A. (2018). The influence of egfr inactivation on the radiation response in high grade glioma. Int. J. Mol. Sci..

[190] Barker F.G., Simmons M.L., Chang S.M., Prados M.D., Larson D.A., Sneed P.K., Wara W.M., Berger M.S., Chen P., Israel M.A. (2001). Egfr overexpression and radiation response in glioblastoma multiforme. Int. J. Radiat. Oncol. Biol. Phys..

[191] Cuneo K.C., Nyati M.K., Ray D., Lawrence T.S. (2015). Egfr targeted therapies and radiation: Optimizing efficacy by appropriate drug scheduling and patient selection. Pharmacol. Ther..

[192] Harada H. (2016). Hypoxia-inducible factor 1-mediated characteristic features of cancer cells for tumor radioresistance. J. Radiat. Res..

[193] Kabakov A.E., Yakimova A.O. (2021). Hypoxia-induced cancer cell responses driving radioresistance of hypoxic tumors: Approaches to targeting and radiosensitizing. Cancers.

[194] Zhang M., Qiu Q., Li Z., Sachdeva M., Min H., Cardona D.M., DeLaney T.F., Han T., Ma Y., Luo L. (2015). Hif-1 alpha regulates the response of primary sarcomas to radiation therapy through a cell autonomous mechanism. Radiat. Res..

[195] Wu C., Luo J. (2016). Long non-coding rna (lncrna) urothelial carcinoma-associated 1 (uca1) enhances tamoxifen resistance in breast cancer cells via inhibiting mtor signaling pathway. Med. Sci. Monit..

[196] Lin Y.J., Shyu W.C., Chang C.W., Wang C.C., Wu C.P., Lee H.T., Chen L.J., Hsieh C.H. (2017). Tumor hypoxia regulates forkhead box c1 to promote lung cancer progression. Theranostics.

[197] Barbagallo C., Caltabiano R., Broggi G., Russo A., Puzzo L., Avitabile T., Longo A., Reibaldi M., Barbagallo D., Di Pietro C. (2020). Lncrna linc00518 acts as an oncogene in uveal melanoma by regulating an rna-based network. Cancers.

[198] Ren Y., Zhu H., Han S. (2020). Linc00518 interference inhibits non-small cell lung cancer by upregulating mir216b-5p expression. Cancer Manag. Res..

[199] Luan W., Ding Y., Ma S., Ruan H., Wang J., Lu F. (2019). Long noncoding rna linc00518 acts as a competing endogenous rna to promote the metastasis of malignant melanoma via mir-204-5p/ap1s2 axis. Cell Death Dis..

[200] Wang H.B., Wei H., Wang J.S., Li L., Chen A.Y., Li Z.G. (2019). Down-regulated expression of linc00518 prevents epithelial cell growth and metastasis in breast cancer through the inhibition of cdx2 methylation and the wnt signaling pathway. Biochim. Biophys. Acta Mol. Basis Dis..

[201] He J., Sun M., Geng H., Tian S. (2019). Long non-coding rna linc00518 promotes paclitaxel resistance of the human prostate cancer by sequestering mir-216b-5p. Biol. Cell.

[202] Chang L., Hu Z., Zhou Z., Zhang H. (2018). Linc00518 contributes to multidrug resistance through regulating the mir-199a/mrp1 axis in breast cancer. Cell. Physiol. Biochem..

[203] Albadari N., Deng S., Li W. (2019). The transcriptional factors hif-1 and hif-2 and their novel inhibitors in cancer therapy. Expert Opin. Drug Discov..

[204] Jones D.T., Harris A.L. (2012). Small-molecule inhibitors of the hif pathway and synthetic lethal interactions. Expert Opin. Ther. Targets.

[205] Chen W., Hill H., Christie A., Kim M.S., Holloman E., Pavia-Jimenez A., Homayoun F., Ma Y., Patel N., Yell P. (2016). Targeting renal cell carcinoma with a hif-2 antagonist. Nature.

[206] Nishizawa Y., Konno M., Asai A., Koseki J., Kawamoto K., Miyoshi N., Takahashi H., Nishida N., Haraguchi N., Sakai D. (2018). Hypoxia stimulates the cytoplasmic localization of oncogenic long noncoding rna linc00152 in colorectal cancer. Int. J. Oncol..

[207] Zhao K., Wang X., Xue X., Li L., Hu Y. (2020). A long noncoding rna sensitizes genotoxic treatment by attenuating atm activation and homologous recombination repair in cancers. PLoS Biol..

[208] Luo L., Zhang J., Tang H., Zhai D., Huang D., Ling L., Wang X., Liu T., Zhang Q., Zhang Z. (2020). Lncrna snord3a specifically sensitizes breast cancer cells to 5-fu by sponging mir-185-5p to enhance umps expression. Cell Death Dis..

[209] Moeng S., Son S.W., Lee J.S., Lee H.Y., Kim T.H., Choi S.Y., Kuh H.J., Park J.K. (2020). Extracellular vesicles (evs) and pancreatic cancer: From the role of evs to the interference with ev-mediated reciprocal communication. Biomedicines.

[210] Guo Z., Wang X., Yang Y., Chen W., Zhang K., Teng B., Huang C., Zhao Q., Qiu Z. (2020). Hypoxic tumor-derived exosomal long noncoding rna uca1 promotes angiogenesis via mir-96-5p/amotl2 in pancreatic cancer. Mol. Ther. Nucleic Acids.

[211] Castellano J.J., Marrades R.M., Molins L., Vinolas N., Moises J., Canals J., Han B., Li Y., Martinez D., Monzo M. (2020). Extracellular vesicle lincrna-p21 expression in tumor-draining pulmonary vein defines prognosis in nsclc and modulates endothelial cell behavior. Cancers.

[212] Hong F., Gao Y., Li Y., Zheng L., Xu F., Li X. (2020). Inhibition of hif1a-as1 promoted starvation-induced hepatocellular carcinoma cell apoptosis by reducing hif-1alpha/mtor-mediated autophagy. World J. Surg. Oncol..

[213] Wu Y., Ding J., Sun Q., Zhou K., Zhang W., Du Q., Xu T., Xu W. (2018). Long noncoding rna hypoxia-inducible factor 1 alpha-antisense rna 1 promotes tumor necrosis factor-alpha-induced apoptosis through caspase 3 in kupffer cells. Medicine.

[214] Hsu H.H., Kuo W.W., Shih H.N., Cheng S.F., Yang C.K., Chen M.C., Tu C.C., Viswanadha V.P., Liao P.H., Huang C.Y. (2019). Foxc1 regulation of mir-31-5p confers oxaliplatin resistance by targeting lats2 in colorectal cancer. Cancers.

[215] Dong X., Zhang Y., Chen X., Xue M. (2020). Long noncoding rna linc00511 regulates the proliferation, apoptosis, invasion and autophagy of trophoblast cells to mediate pre-eclampsia progression through modulating the mir-31-5p/homeobox protein a7 axis. J. Obstet. Gynaecol. Res..

[216] Taniue K., Akimitsu N. (2021). The functions and unique features of lncrnas in cancer development and tumorigenesis. Int. J. Mol. Sci..

[217] Lee H.Y., Son S.W., Moeng S., Choi S.Y., Park J.K. (2021). The role of noncoding rnas in the regulation of anoikis and anchorage-independent growth in cancer. Int. J. Mol. Sci..

[218] Hompland T., Fjeldbo C.S., Lyng H. (2021). Tumor hypoxia as a barrier in cancer therapy: Why levels matter. Cancers.

